# Comparing the success of active and passive restoration in a tropical cloud forest landscape: A multi-taxa fauna approach

**DOI:** 10.1371/journal.pone.0242020

**Published:** 2020-11-10

**Authors:** Juan Manuel Díaz-García, Fabiola López-Barrera, Eduardo Pineda, Tarin Toledo-Aceves, Ellen Andresen

**Affiliations:** 1 Red de Ecología Funcional, Instituto de Ecología, A. C., Xalapa, Veracruz, México; 2 Red de Biología y Conservación de Vertebrados, Instituto de Ecología, A. C., Xalapa, Veracruz, México; 3 Instituto de Investigaciones en Ecosistemas y Sustentabilidad, Universidad Nacional Autónoma de México, Morelia, Michoacán, México; University of Sydney, AUSTRALIA

## Abstract

Tropical forest restoration initiatives are becoming more frequent worldwide in an effort to mitigate biodiversity loss and ecosystems degradation. However, there is little consensus on whether an active or a passive restoration strategy is more successful for recovering biodiversity because few studies make adequate comparisons. Furthermore, studies on animal responses to restoration are scarce compared to those on plants, and those that assess faunal recovery often focus on a single taxon, limiting the generalization of results. We assessed the success of active (native mixed-species plantations) and passive (natural regeneration) tropical cloud forest restoration strategies based on the responses of three animal taxa: amphibians, ants, and dung beetles. We compared community attributes of these three taxa in a 23-year-old active restoration forest, a 23-year-old passive restoration forest, a cattle pasture, and a mature forest, with emphasis on forest-specialist species. We also evaluated the relationship between faunal recovery and environmental variables. For all taxa, we found that recovery of species richness and composition were similar in active and passive restoration sites. However, recovery of forest specialists was enhanced through active restoration. For both forests under restoration, similarity in species composition of all faunal groups was 60–70% with respect to the reference ecosystem due to a replacement of generalist species by forest-specialist species. The recovery of faunal communities was mainly associated with canopy and leaf litter covers. We recommend implementing active restoration using mixed plantations of native tree species and, whenever possible, selecting sites close to mature forest to accelerate the recovery of tropical cloud forest biodiversity. As active restoration is more expensive than passive restoration, both strategies might be used in a complementary manner at the landscape level to compensate for high implementation costs.

## 1 Introduction

The practice of forest restoration is increasing worldwide, in an effort to mitigate the decline of biodiversity and ecosystem services caused by habitat degradation [1]. This global effort to recover degraded ecosystems reflects the declaration by the United Nations General Assembly of 2021–2030 as the “Decade on Ecosystem Restoration” [2]. To achieve forest restoration, ambitious initiatives include the implementation of both passive and active restoration strategies [3]. Nevertheless, passive restoration has been pointed out as the most cost-effective forest restoration strategy for large-scale implementation [4].

Recently, two meta-analyses supported the notion that passive restoration is more successful than active restoration for recovering vegetation structure and animal diversity in tropical forests [5, 6]. However, Reid et al. [7] showed that in both meta-analyses the comparison between passive and active restoration did not account for variations in the land-use history of the sites being compared. To avoid confounding factors, comparisons need to be made in sites where both restoration strategies have been implemented at the same time in the same landscape [7]. Yet, studies using this approach represent <10% of the tropical forest restoration literature [5].

Tropical forest restoration studies that compare active and passive restoration under the same conditions have mainly measured vegetation variables [6]. Faster recovery of vegetation structure (i.e. tree density, basal area, and canopy cover) has been reported for active restoration, but results for species composition have been inconclusive [8–13]. Differences in vegetation recovery between active vs. passive restoration may affect animals [8, 14]. Animals may respond actively to plant recovery [15] and can modulate ecological trajectories by providing important ecosystem functions, such as pollination, seed predation and dispersal, herbivory, and by affecting energy flows [16–18].

Relatively few studies include animals and animal-plant interactions in their assessment of restoration success [15]. Active and passive restoration strategies have been found to be equally successful in recovering species richness, biomass and behavior of different faunal groups [8, 14, 19]. However, recovery of species composition varies depending on restoration strategy and faunal group [14]. Recently, Díaz-García et al. [19] reported that the recovery of required habitat characteristics for threatened amphibians was greater in active vs. passive restoration sites.

Determining which restoration strategy is more effective for recovering fauna may depend on the community attributes being evaluated [15, 19, 20]. For example, species richness tends to recover faster than species composition or abundance [15, 21]. Furthermore, responses to restoration also depend on the taxa being assessed, due to their particular ecological traits, such as habitat specialization [6, 15]. Thus, multi-taxa studies can be useful for assessing the general success of a restoration project.

Our aim was to compare the success of active and passive restoration strategies based on the responses of amphibian, ant, and dung beetle communities in a tropical cloud forest landscape, with emphasis on forest-specialist species. These three groups have different ecological traits, are highly sensitive to modifications in their habitats [22], and perform important ecological functions, such as seed dispersal, insect population control, and nutrient cycling [23–25]. Also they are taxa of conservation concern, as amphibians are the most threatened group of terrestrial vertebrates worldwide [26] and terrestrial insects are undergoing marked population declines [27]. We compared species richness, abundance/occurrence, and community composition of these groups in a 23-year-old actively restored forest, a 23-year-old passively restored forest, a cattle pasture (degraded ecosystem), and a mature cloud forest (reference ecosystem). We also compared the responses of forest-specialist vs. generalist species. Finally, we analyzed the relationships between the recovery of each faunal community and environmental variables. The results of our study strengthen our understanding of the reestablishment of habitat conditions in cloud forests under active vs. passive restoration, which is relevant for the conservation of many animal species inhabiting these threatened ecosystems.

## 2 Materials and methods

### 2.1 Study area

**Fig 1:**
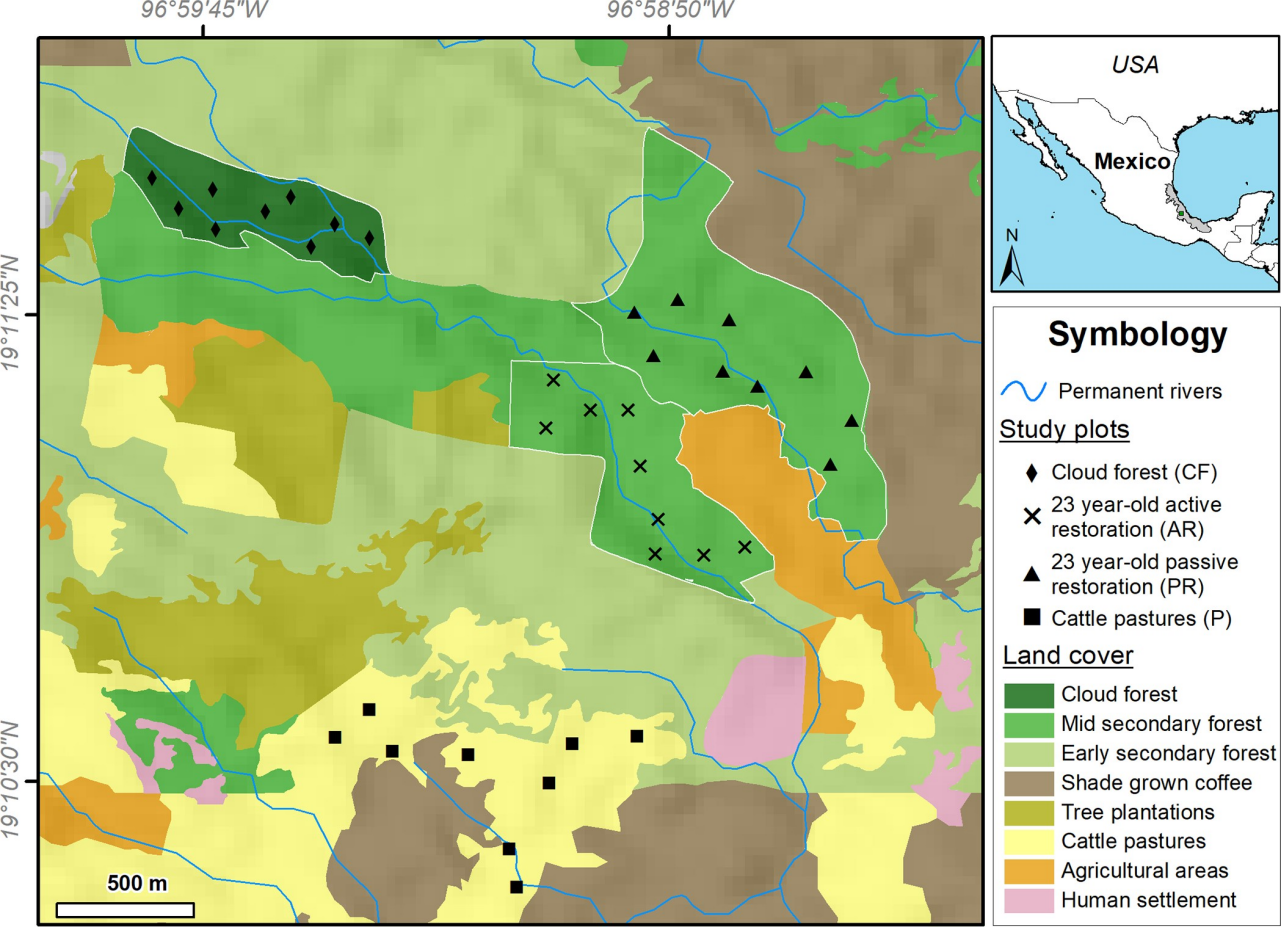
Map showing the study area, land-cover types, and the location of 36 study plots (black markers) in four vegetation types studied in the mountainous region of central Veracruz, Mexico. Tree plantations include monocultures of bamboo, avocado and pine.

This study was carried out in the municipality of Huatusco (19°11’23” N, 96°59’11” W, 1,300 m a.s.l.) in the mountainous region of central Veracruz, Mexico (Fig 1). The climate is sub-humid, with a mean annual temperature of 17.1°C and a mean annual precipitation of 1,850 mm [28]. Huatusco currently has ~ 10,000 ha of cloud forest, but only 30% are mature forest patches. These patches are immersed in a matrix of agricultural crops, agroforestry systems, cattle pastures, and secondary forest [29].

### 2.2 Study sites and spatial design

In 1995, after 25 years of extensive livestock production, the owner and members of the Las Cañadas Cooperative in the municipality of Huatusco, began to restore cloud forest in two patches of cattle pasture. Active restoration was used at a site that was 995 m from a cloud forest remnant. Simultaneously, passive restoration was implemented in a site 1,152 m from the cloud forest and adjacent to active restoration site [13]. We used these two restoration sites in our study and also selected an active pasture and a conserved forest (Fig 1). At the time of our study, the main characteristics of our study sites were:

1. Cattle pasture (degraded ecosystem; P): 80 ha of active pasture (~ 1 head/ha), which was cloud forest prior to transformation about 40 years ago. Tree density is low (mean ± SD: 25 trees/ha ± 41.8; trees ≥ 10 cm diameter at breast height) and mean canopy height is 8 m (± 12.5). Abundant tree species include *Acacia pennatula*, *Psidium guajava*, and *Quercus insignis*, and the ground is covered by exotic grasses (*Axonopus* sp. and *Cynodon* sp.). This site is has a distance of ~1,800 m from the cloud forest.
2. Passive restoration (PR): 62 ha of a 23-year-old forest under passive restoration. This area used to be cattle pasture, but a natural regeneration process began in 1995 after cattle were excluded. Tree density is 350 trees/ha ± 38.1, with a dominance of *Myrsine coriacea*, *Trema micrantha*, and *Quercus sapotifolia* [13]. Canopy is closed, with a mean height of 13.9 m (± 1.2). The ground is mainly covered by herbs, shrubs, leaf litter, and patches of the invasive fern *Pteridium arachnoideum*.
3. Active restoration (AR): 37 ha of 23-year-old restoration plantations. This area was used as cattle pasture until 1995, when a mixture of native tree species (*Fraxinus uhdei*, *Juglans pyriformis*, *Liquidambar styraciflua*, *Platanus mexicana*, *Q*. *sapotifolia* and *Quercus* spp.) were planted (1090 saplings/ha; 1–1.5 m in height) following cattle exclusion. The only additional management activity was manual vegetation clearing around saplings two years after reforestation. Tree density is 462.5 trees/ha ± 31.8, mainly *L*. *styraciflua*, *M*. *coriacea*, *T*. *micrantha*, and *Q*. *sapotifolia* [13]. Canopy is closed, with a mean height of 18.9 m (± 1.1). The ground is mainly covered by herbs, shrubs, and leaf litter.
4. Cloud forest (reference ecosystem; CF): 30 ha of mature cloud forest. Tree density is 614.7 trees/ha ± 50.3, with a dominance of *Q*. *paxtalensis*, *Q*. *lancifolia*, and *Q*. *insignis* [13]. Canopy is closed with average height of 24.5 m (± 1.1). The ground is mainly covered by leaf litter, fallen trunks, herbs, and shrubs.

At the time of our study, all sites were privately owned and all owners granted permission to access their properties and carry out this study. In each vegetation type, we established nine 500 m^2^ plots (50 m × 10 m) with a north-south orientation. The 36 plots were separated from each other and from other vegetation types by a minimum of 100 m. All plots were located between 1,330 and 1,450 m a.s.l.

### 2.3 Faunal communities

For amphibians, we sampled each of the 36 plots on three occasions during the rainy season of 2018: in June, August and October. During each sampling, two people recorded amphibians in each plot at night (between 20:00 and 01:00 h) using visual encounter surveys [30]. We captured all individuals and kept them in separate plastic bags with a bit of the substrate where it was found (i.e. leaf litter or tree leaves). We kept animals in a safe and cool place to later identify them and take morphometric measures. All individuals were released within 24 hours of their capture in the same plot where they were found.

In the case of dung beetles, we sampled once in the dry season (May 2018) and again in the rainy season (September 2018). At three points in each plot (0, 25 and 50 m), we set up two plastic pitfall traps (500 ml) separated by 10 m (the width of the plot). Traps were filled to one quarter of their capacity with soapy water and were protected from the rain with plastic plates. Three different bait types were used: human feces, carrion (tilapia fish that had been decomposing for two days), and a mixture of guava and banana (1:1). We used ~20 g of bait placed in a small plastic cup suspended inside the pitfall traps. Bait type was alternated between pitfall traps from right to left along each plot to avoid that traps with the same type of bait were too close to each other or on the same side of the plot. We checked traps after 72 h and collected all beetles. Specimens were identified to species level in the laboratory.

For ants, we sampled once during the dry season (May 2018) and again in the rainy season (September 2018). All plots were sampled using complementary techniques to include the different microhabitats used by ants. We marked four points along each plot (0, 17, 34 and 50 m), and set up two plastic traps at each point: one (500 ml) buried at ground level (pitfall), and one (200 ml) placed on a tree, 2 m above the ground. Both types of traps were separated by 10 m (the width of the plot). The eight traps per plot were filled to one quarter of their capacity with soapy water and were protected from the rain with plastic plates. Pitfall traps were either not baited, or baited with ~20 g of tuna in a plastic cup inside the trap. Tree traps were baited with either ~20 g of tuna or honey in a plastic cup inside the trap. As for dung beetles, trap and bait types were alternated from right to left along each plot. We checked traps after 72 h and collected all ants. Additionally, in the center of each plot we collected all leaf litter in 1 m^2^ and processed it with Winkler sacks to extract ants [31]. Ants were identified to species level in the laboratory. Samplings of ants and dung beetles were not conducted simultaneously, in both seasons dung beetles were sampled first.

We did not collect any species that categorized as threatened by the IUCN Red List and SEMARNAT-NOM 059 (Mexican government regulation). Collected dung beetles and ants were deposited in the Colección Entomológica of the Instituto de Ecología A. C. (IEXA; Reg. SEMARNAT: Ver. IN.048.0198) following sampling and preservation protocols.

### 2.4 Habitat specialization

We grouped amphibian, dung beetle, and ant species into two categories: (1) forest-specialist species and (2) generalist species. Forest-specialist species are those whose populations thrive better in mature forests and have limited tolerance to environmental changes. Generalist species are those that have a broader environmental tolerance and thriving populations can be found in a variety of natural and modified habitats [32, 33]. To assign species to a category we consulted specialized literature for each group (S1 Appendix). Ants not identified to species level were not categorized.

### 2.5 Environmental variables

In each plot we measured the following environmental variables: distance to the closest permanent stream, distance to the closest cloud forest edge, tree density, number of fallen trunks, soil compaction, epiphyte cover, and cover for the following strata: canopy (understory and canopy trees with general height between 5–20 m), shrub (0.5–1.5 m), and prostrate (< 0.5 m). Distances were measured from the central point of each plot using Google Earth images from 2019.

Between March and September 2018, we counted the number of trees with a diameter at breast height (DBH) ≥ 10 cm in a 200 m^2^ (20 m × 10 m) subplot located in the center of each plot. Along the central line of each plot we marked three points, at 0, 25 and 50 m, to measure canopy cover with a photograph taken 120 cm above the ground and processed with the software Image J [34]. At those same points, we measured soil compaction using a pocket penetrometer, and in a 1 m^2^ quadrat we visually estimated the cover (%) of the prostrate stratum (leaf litter, bare soil, exotic grasses, non-grass herbaceous plants) and the shrub stratum (invasive fern *P*. *arachnoideum*, Piperaceae shrubs, other shrubs). We separated Piperaceae shrubs from other shrubs due to some ant species have close relationships with species of this family. For example, in anthills of *Camponotus sp*. and *Solenopsis sp*., the seeds germinate easily forming ant gardens [35]. As epiphytes can be relevant habitat features for frogs, salamanders and ants, we also visually estimated the percentage of vascular epiphyte cover (%) on the branches and trunk of the tree (≥ 10 cm DBH) closest to each recording point. Finally, we counted the number of fallen trunks (≥ 10 cm DBH) within the whole plot.

### 2.6 Data analysis

To ensure valid comparisons of species richness among vegetation types, we estimated the sample coverage (Ĉ_*n*_) for each vegetation type using the formula [36]:

$${Ĉ}_{n}=1-\frac{f_{1}}{n}[\frac{(n-1)f_{1}}{(n-1)f_{1}+2f_{2}}]$$

Where *f*_*1*_ is the number of singleton species, *f*_*2*_ is the number of doubletons, and *n* is the total number of individuals recorded for amphibians and dung beetles, or the total occurrence-frequency recorded for ants. We calculated total species richness and richness per habitat-specialization category for each faunal group and compared them among vegetation types using their 95% confidence intervals [37]. For these analyses we used the ‘iNEXT’ package [38] in R version 1.1.383 [39].

We estimated the abundance per plot by pooling the number of individuals collected in all samplings for amphibians and dung beetles. To compare abundances between vegetation types, we used generalized linear models (GLM) with a Poisson distribution and the log link function, and *post hoc* tests of contrasts. Since ants exhibit social behavior, we used occurrence-frequency instead of the number of individuals. We calculated the occurrence-frequency of a species as the number of plots in which the species was present and summed the occurrences recorded in each of the two samplings (i.e. maximum occurrence-frequency for a species that was present, in all 9 plots in every sampling was 9 x 2 = 18). We used a goodness-of-fit Chi-square test and a *post hoc* test to compare the occurrence-frequency of ants among vegetation types. For these analyses we used the ‘gmodels’ [40] and ‘fifer’ packages [41] in R.

To compare community composition among vegetation types, we built a dendrogram using the Bray-Curtis similarity index for each animal community. To reduce the influence of the most abundant species, we used the chord transformation on the abundance matrix of amphibians and dung beetles [42]. Then, we ran a permutational multivariate analysis of variance (Permanova; 999 permutations) of Bray-Curtis indexes. We built a heat map using the abundance/frequency of each animal group, and we compared changes in the abundance of each species among the four vegetation types. These analyses were run using the ‘vegan’ [43] and ‘ggplot2’ packages [44] in R.

To explore the relationship between faunal recovery and environmental variables, we built models that included as predictors different combinations of the environmental variables, according to the ecological traits of each faunal group (S2 Appendix). Prior to constructing the models, we eliminated variables that were highly correlated with others (Pearson correlation coefficients ≥ 0.6, p < 0.05; S2 Appendix). For the three groups, predictor variables included in models were: distance to the closest cloud forest edge, tree density, canopy cover, and the covers of invasive fern (*P*. *arachnoideum*), leaf litter, and non-grass herbaceous plants. Models for amphibians also included the distance to the closest stream, number of fallen trunks, and epiphyte cover. Models for dung beetles also included soil compaction. Models for ants also included the number of fallen trunks, epiphyte cover, and soil compaction. Response variables were species richness and abundance (amphibians and dung beetles), or occurrence-frequency (ants) per plot, for all species and per habitat-specialization category.

Selected predictor variables were standardized with the scale function and included in a GLM with Poisson distribution and log link function to determine their relationship to the response variables. We used a backward stepwise process to select the best model using the second order Akaike Information Criterion (AICc) and a threshold value of ΔAICc ≤ 2 for determining equally plausible models. Models were constructed using the ‘gmodels’ [40], ‘MASS’ [45] and ‘geiger’ packages [46] in R.

## 3 Results

### 3.1 Species richness

We found a total of 13 amphibian species (7 forest specialists and 6 generalists), 15 dung beetle species (5 forest specialists and 10 generalists), and 39 ant species (19 forest specialists, 16 generalists and 4 undefined; S1 Appendix). Sample coverage in each vegetation type was > 98% for amphibians, > 99% for dung beetles, and > 90% for ants.

**Fig 2:**
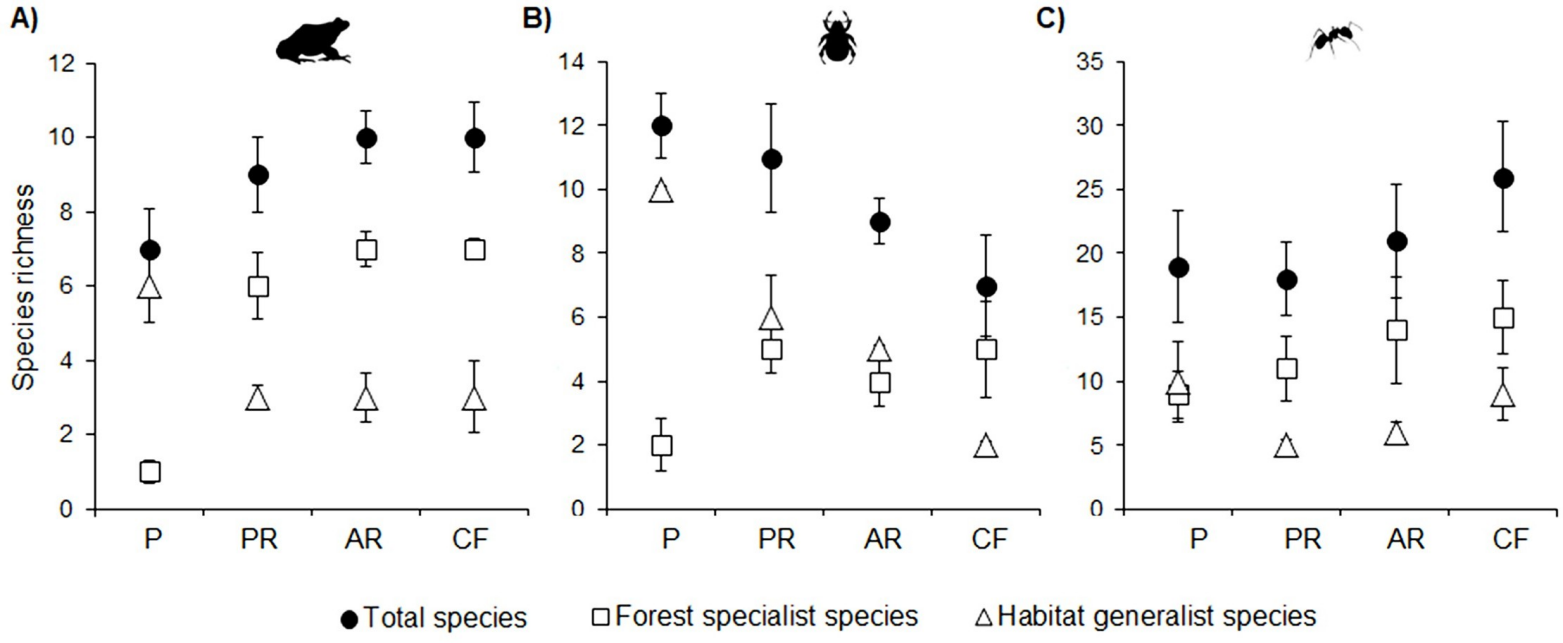
Species richness of amphibians (a), dung beetles (b) and ants (c) in four vegetation types. P = cattle pasture, PR = forest under passive restoration, AR = forest under active restoration and CF = mature cloud forest. Error bars denote 95% confidence intervals.

For amphibians, total richness did not vary significantly between cloud forest and either of the restored forests, but was significantly higher in cloud forest and active restoration than in the cattle pasture. Richness of forest-specialists was significantly higher in the three forests than in the cattle pasture, while richness of generalists was significantly higher in cattle pasture than in the other vegetation types (Fig 2A).

For dung beetles, total richness was significantly higher in passive restoration and in cattle pasture than in cloud forest, but did not vary between restored forests. Richness of forest specialists was significantly higher in the three forests than in cattle pasture, while richness of generalist species was significantly higher in cattle pasture compared to the other vegetation types (Fig 2B).

For ants, total species richness was significantly higher in cloud forest compared to passive restoration, but among the other vegetation types there were no significant differences. Richness of forest specialists was similar in the three forests, but was significantly higher in cloud forest than in cattle pasture. Richness of generalists was higher in cloud forest and cattle pasture than in both restored forests (Fig 2C).

### 3.2 Abundance of amphibians and dung beetles, and occurrence-frequency of ants

**Fig 3:**
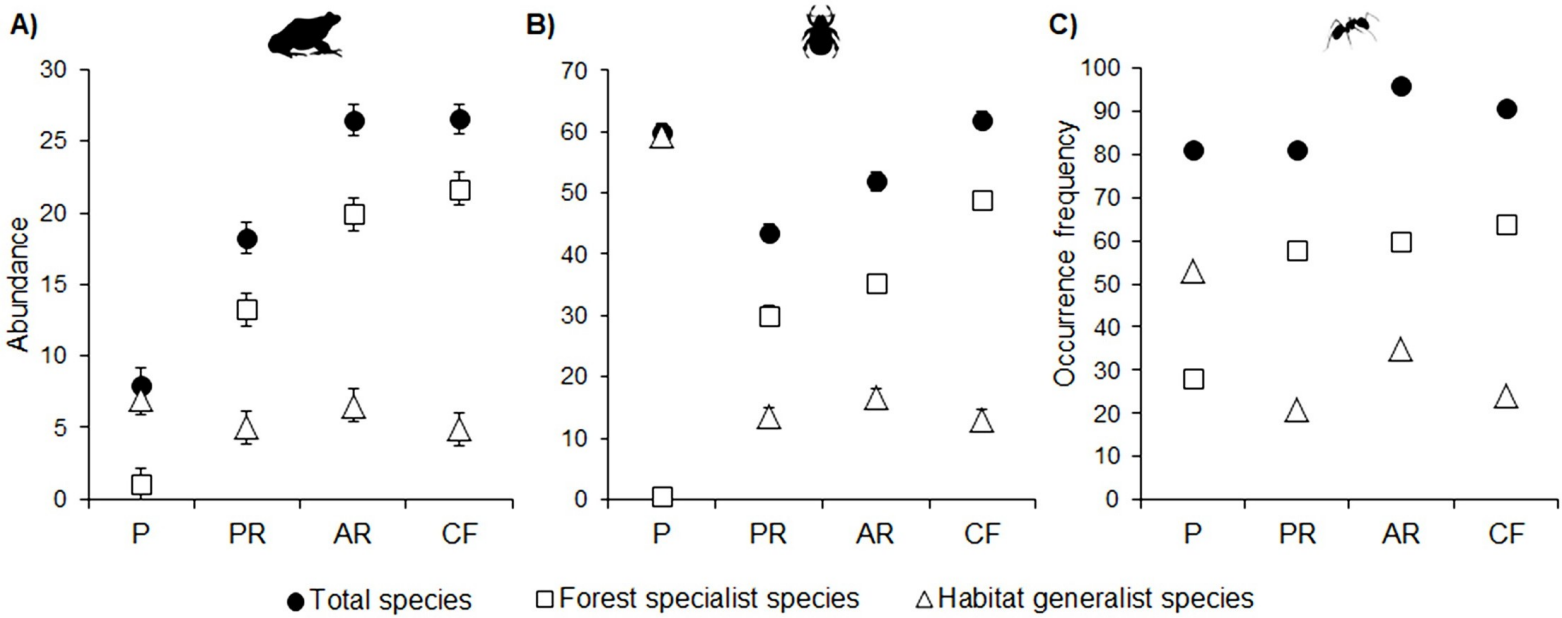
Mean abundance per plot of amphibians (a) and dung beetles (b), and occurrence-frequency of ants (c) in four vegetation types. P = cattle pasture, PR = forest under passive restoration, AR = forest under active restoration and CF = mature cloud forest. Error bars denote standard error. Values of statistical differences among vegetation types are provided in S3 Appendix in (Tables 1 and 2).

Mean abundance of all amphibians and forest specialists was significantly higher in cloud forest and forest under active restoration than in passive restoration or cattle pasture. Abundance of generalist species was similar among all vegetation types (Fig 3A; S3 Appendix).

Mean abundance of all dung beetles was significantly higher in cloud forest and cattle pasture than in both restored forests. Abundance of specialists was higher in cloud forest than in the other vegetation types. Total abundance and abundance of forest specialists were higher in active restoration than in passive restoration. Abundance of generalists was higher in cattle pasture than in the other vegetation types (Fig 3B; S3 Appendix in Tables 1, 2).

Occurrence-frequency of all ants was similar among all vegetation types. Occurrence-frequency of specialist species was higher in cloud forest and in both forests under restoration, compared to cattle pasture. For generalist species, occurrence-frequency was higher in cattle pasture than in the other vegetation types (Fig 3C; S3 Appendix in Tables 1, 2).

### 3.3 Community composition

**Fig 4:**
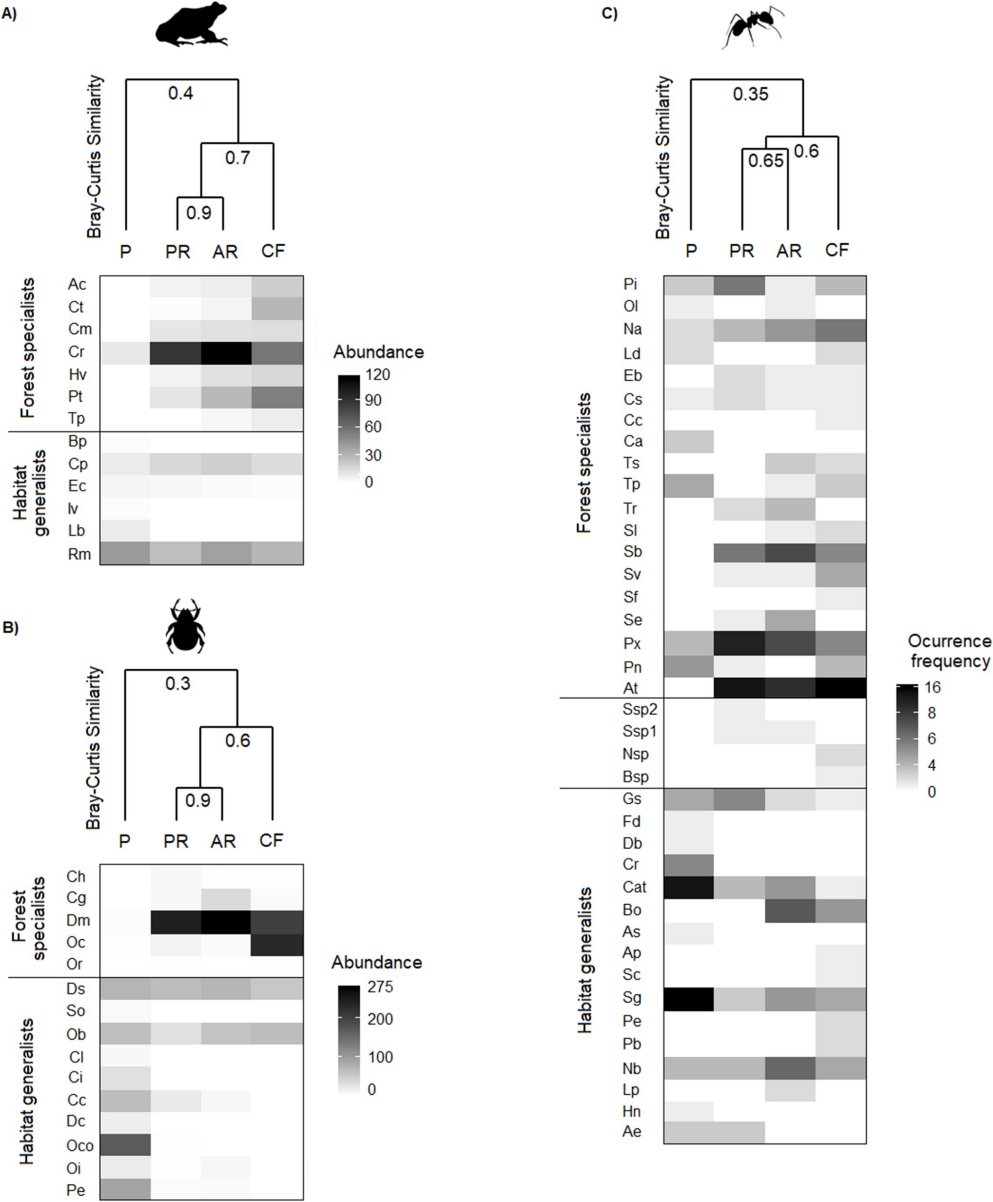
Dendrogram of similarity (Bray-Curtis index) and heatmap based on amphibian abundance (a), dung beetle abundance (b), and ant occurrence-frequency (c) in four vegetation types: P = cattle pasture, PR = forest under passive restoration, AR = forest under active restoration and CF = mature cloud forest. In all dendrograms: 0 = completely different and 1 = completely identical. Species names corresponding to each code are provided in S1 Appendix. Four ant species were only identified to genus level.

We found differences in species composition among vegetation types for amphibians (F = 3.1, *P* = 0.001), dung beetles (F = 28.1, *P* = 0.001), and ants (F = 8.4, *P* = 0.001). For all faunal communities, the highest similarity values were observed between both forests under restoration (65–90%). Species compositions in both forests under restoration were relatively similar to that in cloud forest (60–70%), but less similar when compared to pasture (30–40%; Fig 4).

For dung beetles, none of the forest specialists was found in cattle pasture. For amphibians, only one of the 7 forest specialists was present in pasture. For ants, 9 of the 19 forest specialists were present in pasture. Evenness of amphibian and ant communities was greater in cloud forest than in the other vegetation types. However, the lowest evenness of beetle communities was recorded in cloud forest. In cloud forest we observed the dominance of two forest-specialist amphibians (*Craugastor rhodopis* and *Parvimolge townsendi*), two forest-specialist dung beetles (*Deltochilum mexicanum* and *Onthophagus cyanellus*), and one forest-specialist ant (*Adelomyrmex tristani)*. In forests under restoration we observed the dominance of one forest-specialist amphibian (*C*. *rhodopis*), one forest-specialist dung beetle (*D*. *mexicanum*), and two forest-specialist ants (*A*. *tristani* and *Pheidole xyston*). The dominant species in cattle pasture were one generalist amphibian (*Rheohyla miotympanum*), one generalist dung beetle (*Onthophagus corrosus*), and two generalist ants (*Solenopsis geminata* and *Camponotus atriceps*; Fig 4).

### 3.4 Relationship between faunal recovery and environmental variables

The most important environmental variables (P <0.001) related to fauna communities varied according to the fauna group, the community attribute, and the type of habitat specialization. For amphibians, species richness of total and forest-specialist species was positively related with canopy cover. Total abundance decreased with greater distance to streams, and increased mainly with higher covers of leaf litter and non-grass herbaceous covers. Abundance of forest-specialists amphibians increased with canopy, leaf litter, and non-grass herbaceous cover. Abundance of generalist amphibians decreased with greater distance to stream, and increased as there was a higher tree density and leaf litter cover (Table 1).

**Table 1:** Results of the generalized linear models (ΔAICc ≤ 2) that best explain species richness, abundance or occurrence-frequency per plot of amphibians, dung beetles and ants (*D* = deviance, *AIC* = Akaike Information Criterion).

	**Environmental variables**									
	DS	DCF	TD	CC	LLC	NG	PC	EC	FT	SC
**AMPHIBIANS**										
**Species richness**										
Total (*D = 53*.*3*, *AIC = 141*.*4*)				↗**				↗*		
Specialist (*D = 62*.*9*, *AIC = 113*.*7*)				↗**	↗*			ns		
Generalist (*D = 28*.*6*, *AIC = 105*.*8*)				ns						
**Abundance**										
Total (*D = 132*.*1*, *AIC = 298*.*9*)	↘**	↘*			↗**	↗**			↗*	
Specialist (*D = 326*.*4*, *AIC = 202*.*7*)	ns			↗**	↗**	↗**				
Generalist (*D = 258*.*1*, *AIC = 254*)	↘**		↘**	ns	↘**					
										
**DUNG BEETLES**										
**Species richness**										
Total (*D = 20*.*9*, *AIC = 147*.*8*)				↘**						
Specialist (*D = 41*.*7*, *AIC = 105*.*8*)				↗**			ns			
Generalist (*D = 51*.*5*, *AIC = 131*.*3*)		↗*		↘*						
**Abundance**										
Total (*D = 539*.*2*, *AIC = 615*.*9*)		↘**	↗**	↘**	↗**	↗**	↘**			↗**
Specialist (*D = 847*.*3*, *AIC = 393*)		↘**	↗**	ns	↗**	↗**	ns		↗**	
Generalist (*D = 776*.*7*, *AIC = 502*)		↗**		↘**	↗*	↗*	↘**			
										
**ANTS**										
**Species richness**										
Total (*D = 23*.*5*, *AIC = 169*)					↗*					ns
Specialist (*D = 27*.*1*, *AIC = 147*.*9*)				↗**						
Generalist (*D = 28*.*3*, *AIC = 131*.*9*)			↘**							
**Occurrence-frequency**										
Total (*D = 22*.*3*, *AIC = 177*.*4*)							ns		↗**	↗*
Specialist (*D = 28*.*6*, *AIC = 161*.*9*)				↗**			ns		↗*	↗*
Generalist (*D = 32*.*8*, *AIC = 142*.*4*)			↘**							

Environmental variables included in the models were: DS = distance to the closest permanent stream, DCF = distance to the closest cloud forest edge, TD = tree density, CC = canopy cover, LLC = leaf litter cover, NG = cover of non-grass herbaceous plants, PC = *Pteridium arachnoideum* cover, EC = epiphyte cover, FT = number of fallen trunks and SC = soil compaction. ↗ = positive relationship, ↘ = negative relationship

* *P* < 0.05

** *P* < 0.01, ns = not significant.

For dung beetles, total species richness decreased but forest-specialist richness increased with canopy cover. Abundance of total and forest-specialist species increased mainly in sites with higher tree density and greater covers of leaf litter and non-grass herbaceous plants, but decreased with distance to cloud forest. Conversely, abundance of generalist species increased with distance to cloud forest, and in sites with greater cover of leaf litter and non-grass herbaceous plants (Table 1).

For ants, forest-specialist richness and occurrence-frequency were positively related with canopy cover. Occurrence-frequency of total species was higher as the number of fallen trunks increased. Species richness and abundance of generalist ants decreased with higher tree density (Table 1).

## 4 Discussion

In this study, the active and passive restoration sites had similar land-use histories and shared the same landscape matrix, and thus had a similar probability of arrival of flora and fauna. Therefore, we were able to compare both restoration strategies in terms of the recovery of three faunal communities. Overall, our study shows that after 23 years of implementing passive and active cloud forest restoration in one tropical landscape, the recovery of species richness and community composition of the three animal taxa was similar for both restoration strategies. However, active restoration promoted a greater recovery of forest-specialist species in the three groups, resulting in a greater abundance of these species. This result emphasizes the important role that active restoration can play in mitigating the impact of habitat loss and degradation on the most vulnerable species, and in recovering ecological functions relevant to forest integrity. Even so, after more than two decades, neither of the restoration strategies was successful in fully recovering the abundance or community composition of amphibians, dung beetles, and ants found in the mature cloud forest. This highlights the need to conserve and protect mature forests, particularly near restoration sites.

### 4.1 Faunal recovery: Active *versus* passive restoration

Active and passive restoration were equally successful in recovering species richness for all three animal groups, which coincides with results of previous comparisons between these two strategies for amphibians, reptiles, mammals, birds [14], and arthropods [47]. However, species richness can be less informative than other community attributes when assessing restoration progress [48, 49]. We found that community composition in restored forests had a similarity of ~63% to the faunal communities of cloud forest. Studies have shown that, as time progresses, species composition of ants [48], dung beetles [50], amphibians [51], mammals [21], and birds [52] in restoration sites approximates that of reference forests.

Our results also show that the recovery of forest-specialist species can be a useful variable for measuring restoration success [1, 53]. Recovery of forest specialists was a sensitive metric, as it allowed us to differentiate the effectiveness of active vs. passive restoration. Previous studies have recorded the recovery of tropical forest specialists with both active [50, 54, 55] and passive restoration [49, 56]. However, the recovery of forest-specialist species has rarely been compared for the two restoration strategies implemented in the same landscape. In this study, active restoration in a mixed native plantation promoted a higher abundance/occurrence of forest specialists in the three faunal groups, compared to the area being restored through natural regeneration. Mixed plantations can lead to a greater recovery of vegetation structure than passive restoration does [8–13], and thus promotes faster recovery of the environmental characteristics and resources that forest specialists require, such as prey availability, reproduction sites or relatively humid conditions [57]. Despite this recovery, our active restoration forest had not yet reached the faunal community composition and evenness found in mature cloud forest. Thus, some resources, ecological interactions, and habitat features are still necessary for forest-specialist species to recover in our restoration forests.

### 4.2 Faunal recovery and environmental variables

Of the environmental variables related to the recovery of forest-specialist species of amphibians, dung beetles, and ants, the most important ones were canopy, leaf litter and non-grass herbaceous covers. A closed canopy, a dense leaf litter stratum and the presence of herbs reduce temperature variation and maintain relative humidity, favoring the persistence of forest-specialist animals [50]. Proximity to mature forests was relevant for recovering the abundance of amphibians and forest-specialist dung beetles. It has been stated that conserved forests adjacent to the restoration areas can act as source pools, catalyzing the recovery of animals with dispersal limitation and high sensitivity to changes in their habits, such as forest-specialist ants, dung beetles, and amphibians [19, 48, 50].

Previous studies have found that the recovery of forest-specialist animals in restoration sites is affected by habitat and landscape variables, such as the amount of surrounding forest [15], matrix complexity, temperature, humidity, and competition [56], as well as the recovery of interacting faunal groups. As an example of the latter, dung beetle recovery in restored sites may depend on mammal recovery, which provide their main feeding and nesting resource [50]. In our study, different environmental variables were relevant depending on the community attribute, taxonomic group or forest specialization category. For example, proximity to water bodies is a primary requirement for amphibians with aquatic larvae [19, 51], while fallen trunks and dead wood can benefit various groups of invertebrates [58].

The recovery of vegetation structure (e.g. tree density, basal area and canopy height) in the forests under passive restoration was slower and less homogeneous than that of the forest under active restoration [13, 19]. For example, with active restoration, 44% of the basal area present in the cloud forest was recovered, while with passive restoration only 26% was recovered. In addition, in the forest with passive restoration, there are canopy gaps with 12 times more cover of the invasive fern *Pteridium arachnoideum*, compared to the forest with active restoration [13]. *P*. *arachnoideum* creates a dense layer of vegetation on the ground and prevents the establishment of cloud forest tree species [59]. This slows down succession, and consequently the recovery of forest-specialist animals.

In the landscape we studied, cattle pasture plots were further from cloud forest (~1800 m) and streams (~182 m) than restoration forests (~1000 m and ~ 85 m, respectively). Therefore the impoverished faunal communities observed in cattle pastures should be interpreted with caution. Conversely, and most importantly for our conclusions on active vs. passive restoration, both restoration forests had similar distances to cloud forest and streams. As restoration projects are becoming more ubiquitous, future studies will have more opportunities to evaluate the effect of different landscape configurations and to include true replicas of different restoration strategies to be able to make broader inferences.

### 4.3 Management and conservation implications

In the active and passive restoration forests, three ecologically and phylogenetically contrasting faunal groups (amphibians, dung beetles, and ants) recovered partially (70–100% of species richness; 69–100% of abundance; 60–70%species composition) after 23 years. Importantly, this recovery included forest-specialist species, suggesting that both restoration forests are following the desired trajectory of faunal succession, though apparently at a faster rate in the actively restored forest.

Based on the relationships we found between environmental variables and faunal recovery, we suggest that in either restoration type, additional actions can be implemented to accelerate the recovery of amphibians, ants and dung beetles. Canopy closure can be enhanced by planting broad-leaved species with fast crown expansion. A mixture of evergreen and semi-deciduous tree species would also maintain a balance between canopy cover and a dense layer of leaf litter that favors faunal communities. Also, restoration forests can be enriched and succession accelerated by planting trees in canopy gaps, which are frequently dominated by grassy or invasive species. Piles of dead wood of different native species can also be added during early restoration stages to increase the abundance of insects and other invertebrates thus favoring nutrient cycling ecological in the restored forests. Finally, the creation of artificial ponds in early restoration stages, particularly in sites that are not adjacent to streams, can favor the persistence and colonization of amphibians. Amphibians are of great conservation concern, especially in the Neotropics where land use change, forest degradation and emergent diseases such as the chytrid fungus, have severely decimated their populations [60].

The decision of which restoration strategy (active vs. passive) should be used depends on project goals, financial resources, the presence of reference ecosystems, and the land use history of the site [61]. Passive restoration requires less economic investment than active restoration, which can be a decisive factor [4]. Therefore, in landscapes with a relatively high degree of forest cover, passive and active restoration sites may function as complementary strategies for recovering biodiversity and combining both might help to mitigate implementation costs [13]. However, restoration efforts will be futile if the remnants of mature forest and the forests under restoration are not effectively managed and protected.

## Supporting information

S1 File (PDF)

S1 Appendix Species of amphibians, ants and dung beetles recorded in four vegetation conditions, their abundance or occurrence-frequency, and habitat specialization type. P = cattle pasture, PR = forest under passive restoration, AR = forest under active restoration and CF = mature cloud forest.
(DOCX)

S2 Appendix Environmental variables in the four vegetation conditions studied: P = cattle pasture, PR = 23-year-old forest under passive restoration, AR = 23-year-old forest under active restoration and CF = mature cloud forest. Mean and standard deviation (in parentheses) values are presented for all variables. Environmental variables considered in models to predict responses of each group are indicated with an X. NC = not considered in models due to high Pearson correlation coefficients (≥ 0.6, p < 0.05).
(DOCX)

S3 Appendix Table 1. Results of generalized linear models used to compare the abundance of amphibians and dung beetles, and results of goodness-of-fit Chi-square tests used to compare the occurrence-frequency of ants. P = cattle pasture, PR = 23-year-old forest under passive restoration, AR = 23-year-old forest under active restoration and CF = mature cloud forest. Table 2. Results of post hoc tests comparing the abundance of amphibians and dung beetles, and the occurrence-frequency of ants. P = cattle pasture, PR = 23-year-old forest under passive restoration, AR = 23-year-old forest under active restoration and CF = mature cloud forest. In all comparisons the degrees of freedom = 1.
(DOCX)
