# Supplementary material for: Comparing the success of active and passive restoration in a tropical cloud forest landscape: A multi-taxa fauna approach
Source: PLoS One. 2020 Nov 10;15(11):e0242020. doi: 10.1371/journal.pone.0242020 (PMC7654786; doi:10.1371/journal.pone.0242020)
Supplement: S1 File — (PDF) [file pone.0242020.s001.pdf]

Biological Conservation 242 (2020) 108400

Contents lists available at ScienceDirect

Biological Conservation

journal homepage: www.elsevier.com/locate/biocon

Does forest restoration assist the recovery of threatened species? A study of cloud forest amphibian communities

J.M. Díaz-García[a,b], F. López-Barrera[b], T. Toledo-Aceves[b], E. Andresen[c], E. Pineda[a,*]

[a] Red de Biología y Conservación de Vertebrados, Instituto de Ecología, A.C. Carretera Antigua a Coatepec No. 351, El Haya, 91000 Xalapa, Veracruz, Mexico
[b] Red de Ecología Funcional, Instituto de Ecología, A.C. Carretera Antigua a Coatepec No. 351, El Haya, 91000 Xalapa, Veracruz, Mexico
[c] Instituto de Investigaciones en Ecosistemas y Sustentabilidad, Universidad Nacional Autónoma de México, Antigua Carretera a Pátzcuaro No. 8701, Ex Hacienda de San José de la Huerta, 58190 Morelia, Michoacán, Mexico

ARTICLE INFO

Keywords:
Active restoration
Fauna recovery
Passive restoration
Tropical forest
Secondary forest
Mexico

ABSTRACT

Forest restoration may support recovery and conservation of biodiversity. However, the response of biodiversity to forest restoration is likely to vary depending on the restoration strategy used and taxa considered. Our goal was to assess the recovery of amphibians, a highly threatened biological group, in three cloud forests under different restoration strategies. We compared different measures of species diversity in cattle pasture, 13-year-old forest under passive restoration (P13), 23-year-old forest under passive restoration (P23), 23-year-old forest under active restoration (A23) and mature cloud forest in Mexico. We sampled amphibians in 45 plots and measured landscape and habitat variables to assess their influence on amphibian recovery. We found a total of 13 amphibian species, of which 23% are in the Vulnerable (VU) category of the IUCN Red List and 15% are Critically Endangered (CR). All forests under restoration recovered amphibian species richness and composition, including that of the threatened species, but abundance differed among restoration strategies. Abundance of VU and CR species was higher in A23 than in P13 and P23, but that of CR species was highest in cloud forest. Responses to forest restoration differed among taxa; recovery of salamanders was lower than that of anurans. Proximity to water bodies, as well as high canopy and leaf litter cover, had a strong positive influence on amphibian recovery. Our results indicate that recovery of threatened biodiversity could be promoted through forest restoration, particularly active restoration, and highlight the essential role of mature forest in the maintenance and recovery of amphibian communities.

1. Introduction

Ecosystem degradation and loss are considered the greatest threats to biodiversity worldwide (Primack, 2010; IPBES, 2019). Of all the terrestrial vertebrates, the amphibians are the most threatened group (Vié et al., 2009), since 31% of the species are currently at risk of extinction (IUCN, 2019). Tropical forest conversion to anthropized environments is of particular concern (3.6 million ha of primary forest were lost in 2018; WRI, 2019) due to the fact that these forests host > 70% of the world's amphibian species (Bishop et al., 2012). The conservation status of amphibians is even more critical in the Neotropical region, since their populations have declined at a greater proportion than in other regions of the planet (Stuart et al., 2004; Bishop et al., 2012; WWF, 2018). Moreover, amphibian species diversity is not well represented in protected natural areas worldwide (Nori et al., 2015).

Neotropical forest restoration has been recognized as a practice that can contribute to the recovery and conservation of amphibians (Hilje and Aide, 2012; Díaz-García et al., 2017). Through reestablishment of vegetation cover and structure, it is possible to recover the resources and/or conditions necessary for amphibians to recolonize, feed, reproduce and find refuge (Thompson and Donnelly, 2018). Recovery of these processes can depend on the type of restoration strategy implemented; i.e., whether restoration is passive (natural succession) or active (multispecific plantations with native species; Crouzeilles et al., 2017; Trujillo-Miranda et al., 2018). However, there is no consensus regarding which restoration strategy is the most efficient in terms of biodiversity recovery, mainly due to the paucity of studies that have compared the ecological results of both strategies in the same ecosystem type, and because most restoration studies have focused on vegetation recovery while few have addressed fauna (Crouzeilles et al., 2017; Reid et al. 2018).

* Corresponding author.
E-mail address: eduardo.pineda@inecol.mx (E. Pineda).

https://doi.org/10.1016/j.biocon.2019.108400
Received 11 October 2019; Received in revised form 10 December 2019; Accepted 26 December 2019
0006-3207/ © 2019 Elsevier Ltd. All rights reserved.

In addition to the restoration strategy implemented, habitat and landscape characteristics can influence amphibian recovery during Neotropical forest restoration. At landscape level, some important variables include the proximity to remnants of conserved forest, proximity to water bodies (Ficetola et al., 2008; Hernández-Ordoñez et al., 2015) and the complexity of the matrix (Thompson and Donnelly, 2018). At habitat level, amphibian recovery can be determined by the reestablishment of characteristics such as a closed canopy, high leaf litter cover, constant moderate cool temperatures and high levels of environmental humidity (Hilje and Aide, 2012; Díaz-García et al., 2017; Thompson and Donnelly, 2018).

It has been suggested that amphibian recovery can vary among species in Neotropical forests under restoration (Ficetola et al., 2008) and appears to follow a non-random pattern. Phylogenetically related species can respond similarly to modification of their habitat, since they share morphological, physiological or behavioral traits that confer them a similar capacity for dispersion and/or similar habitat requirements (Nowakowski et al., 2018; Pyron, 2018). It has been reported that arboreal amphibians increase in number as woody vegetation recovers (Ríos-López and Aide, 2007), and ground-dwelling amphibians similarly increase with the reestablishment of leaf litter cover (Heinen, 1992). Recovery of lungless salamanders may be determined by the return of leaf litter cover and increased density of fallen logs (Ash, 1997; Welsh and Hodgson, 2013), but the presence of high humidity and lower constant temperatures seems to be more important (Welsh and Hodgson, 2013), because these organisms require those habitat conditions to respire through their skin (Wake and Lynch, 1976; Homyack et al., 2011). Thus, it can be expected that forest restoration will differentially influence amphibians depending on species identity, the particular restoration strategy implemented and the environmental characteristics of the restoration areas.

In the Neotropics, studies of amphibian recovery in forests under restoration are limited compared to those of other vertebrates, such as birds or mammals, and have mainly focused on systems under passive restoration (Crouzeilles et al., 2017). In general, amphibian richness has been found to be the first community attribute to recover with restoration (Ríos-López and Aide, 2007; Hilje and Aide, 2012; Basham et al., 2016), while abundance recovers more slowly (Herrera-Montes and Brokaw, 2010; Hernández-Ordoñez et al., 2015). On the other hand, the species composition of restoration areas generally differs from that of the reference ecosystem (Thompson and Donnelly, 2018), even in secondary forests that have been under passive restoration for > 20 years (Hernández-Ordoñez et al., 2015; Basham et al., 2016). This occurs due to the persistence of generalist species associated with anthropized environments (Ríos-López and Aide, 2007; Díaz-García et al., 2017).

Of the different forest types, the cloud forest is one of the most threatened worldwide (Scatena et al., 2010; Aide et al., 2010, Toledo-Aceves et al., 2011). Restoration of this ecosystem is urgently required in order to recover its characteristic high biodiversity (Challenger, 1998; Álvarez-Aquino et al., 2004; Ramírez-Soto et al., 2018), including a high number of amphibian species and endemisms (Gual-Díaz and Mayer-Goyenechea, 2014). In the last decade, there has been a rapid increase in the number of studies describing the recovery of cloud forest vegetation through passive and active restoration processes (see Williams-Linera, 2015), but few studies consider the fauna recovery (Aide et al., 2010). Amphibian recovery in cloud forest restoration has been evaluated under processes of passive (Basham et al., 2016) and active restoration (Díaz-García et al., 2017), and presented the same species richness but different species composition compared to mature forest. It is still not known how the amphibian recovery varies among different cloud forest restoration strategies, and among groups of species with different characteristics. It is also unknown which environmental variables could have the greatest influence on amphibian recovery under different cloud forest restoration processes.

In order to address these information gaps, the main objective of our study was to evaluate the recovery of amphibian species diversity in three cloud forests under different restoration strategies, comparing these to degraded (cattle pasture) and conserved (cloud forest) ecosystems. Likewise, we examined the response to restoration as a function of taxonomic order, conservation status and habit type. Finally, we evaluated the relationships between amphibian recovery and some landscape and habitat variables. We put particular emphasis on threatened species recovery according to the Red List of Threatened Species of the International Union for Conservation of Nature (IUCN, 2019) because it determines the species that more urgently require protection or rescue worldwide. In addition, using the Red List classification, it is possible to assess the importance of forest restoration and the particular restoration strategy used to recover threatened species (Stuart et al., 2004; Gascon et al., 2007). The results of our study contribute to the conservation of Neotropical amphibian communities by generating particular recommendations for forest restoration practices that promote the recovery of threatened species in anthropized landscapes.

2. Material and methods

2.1. Study area

The study area is located in the municipality of Huatusco (19°11′23″ N, 96°59′11″ W) in the mountainous region of central Veracruz, in southern Mexico (Fig. 1). The climate is sub-humid throughout the year, with three well-defined seasons: a dry cool season (November–March), a dry warm season (April–May) and a wet warm season (June–October; Williams-Linera, 2002). Mean annual precipitation is 1850 mm and mean annual temperature is 19.8 °C (Comisión Nacional del Agua; CONAGUA unpublished data). This region features remnants of conserved cloud forest immersed in a matrix of agricultural crops, shade coffee plantations, cattle pastures (CONABIO, 2010) and cloud forests under restoration.

2.2. Study sites

Based on the results of Trujillo-Miranda et al. (2018), and on the characteristics and history of the study area, we selected five vegetation types: three forests under restoration, one reference ecosystem (cloud forest) and one degraded ecosystem (cattle pasture). Below, we describe these vegetation types (Fig. 1):

1. Cattle pasture (degraded ecosystem; CP): 80 ha of cattle pastureland (~1 head/ha), which had been cloud forest prior to transformation. This site has been pastureland for at least 30 years. This vegetation type features isolated trees (mean ± SD: density 25 trees/ha ± 41.8; trees ≥ 10 cm in diameter at breast height, basal area 4.1 m^2/ha ± 8.3; height 8 m ± 12.5) of the species *Acacia pennatula*, *Psidium guajava* and *Quercus insignis*. The ground level is dominated by a stratum (15 cm in height) of exotic grasses of the genera *Axonopus* and *Cynodon*.
2. 13-year-old forest under passive restoration (P13): 100 ha of secondary forest after 13 years of passive recovery. These areas had been cattle pastures, but a process of natural regeneration began in 2005 with a reduction in the density of cattle (0.15 heads/ha). The density of trees is 289 trees/ha ± 172, with basal area of 11.1 m^2/ha ± 9.7 and height of 13.5 m ± 2.6. The dominant tree species are *A. pennatula*, *Myrsine coriacea* and *Quercus paxtalensis*. The canopy is discontinuous and the soil is covered with leaf litter, exotic grasses and/or the invasive fern *Pteridium arachnoideum*.
3. 23-year-old forest under passive restoration (P23): 62 ha of secondary forest after 23 years of passive recovery. These areas had been cattle pastures but a process of natural regeneration began in 1995 with total exclusion of the cattle. The density of trees is 350 trees/ha ± 38.1, with basal area of 11.8 m^2/ha ± 1.3 and height of 13.9 m ± 1.2. The dominant tree species are *M. coriacea*,

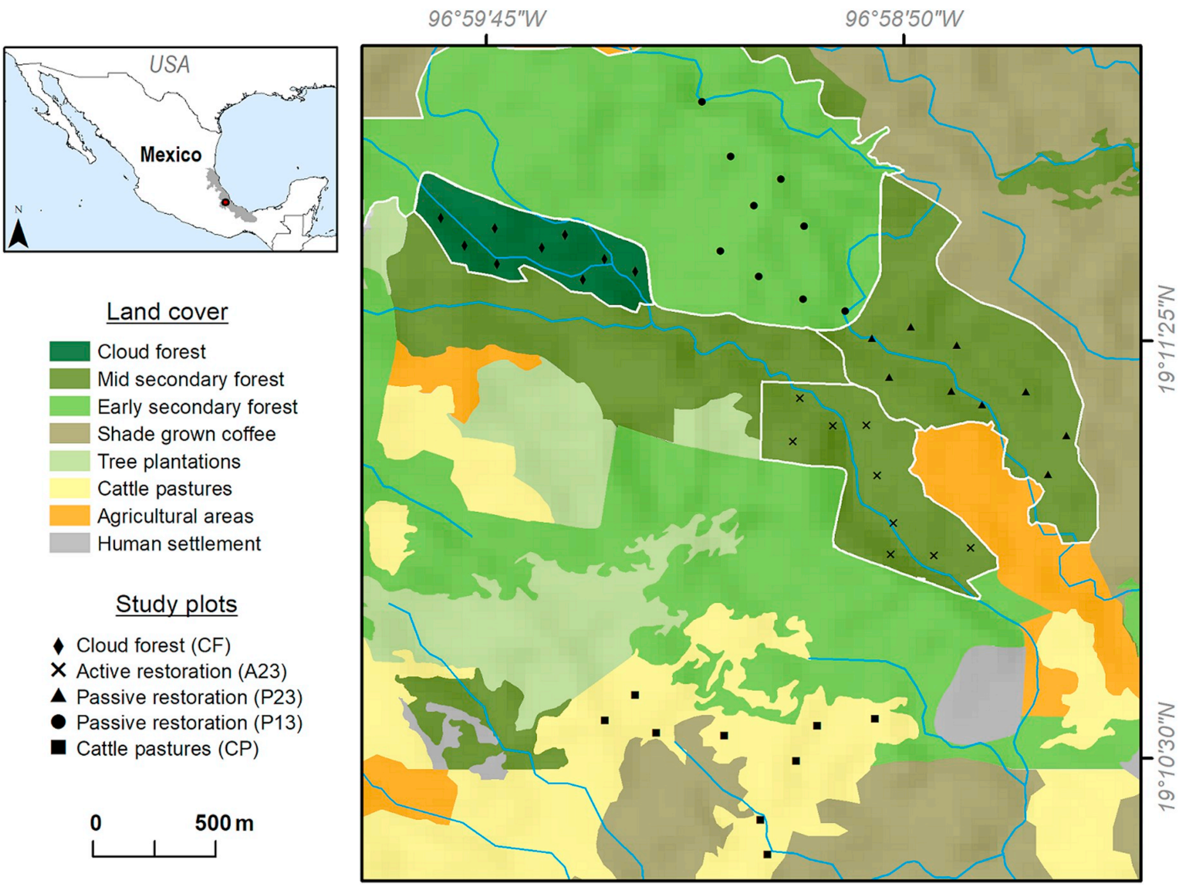

Fig. 1. Location of the 45 study plots (black markers) in five vegetation types studied in the mountainous region of central Veracruz, Mexico. Tree plantations include monoculture plantations of bamboo, avocado and pine. The blue lines denote permanent streams. (For interpretation of the references to color in this figure legend, the reader is referred to the web version of this article.)

Trema micrantha and *Quercus sapotifolia* (Trujillo-Miranda et al., 2018). The canopy is closed and the ground is mostly covered by leaf litter and herbaceous plants, with some presence of *P. arachnoideum*.

4. 23-year-old forest under active restoration (A23): 37 ha of 23-year-old restoration plantations. These areas had been cattle pastures; in 1995, cattle were excluded and mixed plantations were established using 50,000 trees of native species (see Romero-González, 2018). Manual clearing around planted seedlings was conducted on one occasion two years after reforestation in order to reduce competition with exotic grasses. No subsequent management was implemented in the following years. Tree density is 462.5 trees/ha ± 31.8, with basal area of 19.7 m^2/ha ± 2.1 and height of 18.9 m ± 1.1. Dominant tree species are *L. styraciflua*, *M. coriacea*, *T. micrantha* and *Q. sapotifolia* (Trujillo-Miranda et al., 2018). The canopy is closed and leaf litter and herbaceous plants mainly cover the ground.
5. Cloud forest (reference ecosystem; CF): 30 ha remnant of conserved forest. The density of trees is 614.7 trees/ha ± 50.3, with basal area of 44.2 m^2/ha ± 4.7 and height of 24.5 m ± 1.1. The dominant tree species are *Q. paxtalensis*, *Quercus lancifolia* and *Q. insignis* (Trujillo-Miranda et al., 2018). The canopy is closed and the ground is mainly covered by leaf litter. The trees carry high loads of epiphytes.

2.3. Study design

In each vegetation type, we set up nine 500 m^2 plots (50 × 10 m) with north-south orientation and separated among themselves, and from other adjacent vegetation types, by a minimum distance of 100 m. The 45 plots were located between 1330 and 1450 m a.s.l. (Fig. 1).

2.4. Sampling of amphibians

We conducted three samplings of amphibians in June, August and October of 2018. During each sampling, all plots were sampled (in random order) at night (between 20:00 and 01:00 h) by two people, using a visual encounter survey (Crump and Scott, 1994). We identified captured individuals to species level and released them after 24 h at the same site in which they had been captured. The sampled area in each vegetation type was 4500 m^2 (500 m^2 × 9 plots).

2.5. Conservation status

We determined the conservation status of the amphibian species according to the Red List of Threatened Species of the IUCN (2019). We considered as threatened species those listed under the categories of Vulnerable (VU), Endangered (EN) and Critically Endangered (CR), and as non-threatened species those under the categories of Least Concern (LC) and Near Threatened (NT). Species that have not been evaluated by the IUCN were considered as Not Included.

2.6. Species habits

To determine the habits of the species, we consulted specialized literature (Duellman and Trueb, 1986; Wake and Lynch, 1976) and the website AmphibiaWeb (2019). We classified the amphibians into the following habit types: a) terrestrial habit; species that live in the soil,

leaf litter and fallen trunks; b) shrub/herbaceous habit; species that live on vegetation at heights < 2 m; c) arboreal habit; species that live on vegetation at heights > 2 m, and d) semiaquatic habit; species that inhabit both water bodies and terrestrial habitats.

2.7. Landscape and habitat variables

We considered two landscape variables: distance to the closest permanent stream and distance to the closest cloud forest edge. We calculated these distances from the central point of each plot using Google Earth images (2019) and the software ArcMap.

We considered the following habitat variables: tree density, height and basal area, number of fallen trunks, and the canopy, epiphyte, shrub and prostrate stratum covers. Between March and September 2018, we counted the number of adult trees ≥ 10 cm in diameter at breast height (DBH) and measured their height and DBH in subplots of 200 m^2 (20 × 10 m) located in the center of the plots. Along the central line of each plot, we marked three points (0, 25 and 50 m) at which we measured canopy cover with a photograph taken at height 120 cm above ground level and processed with the software Image J, following the method proposed by Korhonen et al. (2006). At these same three points, in a 1 m^2 quadrat, we visually estimated the cover of the prostrate stratum (% leaf litter, % bare soil, % exotic grasses, % non-grass herbaceous plants) and the shrub cover (% fern *P. arachnoideum*, % shrubs). We also visually estimated the percentage of vascular epiphyte cover on the branches and trunk of the tree ($\geq$10 cm DBH) closest to each recording point. Finally, we counted the number of fallen trunks ($\geq$10 cm DBH) within the whole plot.

2.8. Data analysis

2.8.1. Species richness, number of common species and number of dominant species

To ensure valid comparisons of the Hill numbers among the vegetation types, we calculated sample coverage ($\hat{C}_n$) for each vegetation type using the formula:

$$\hat{C}_n = 1 - \frac{f_1}{n}\left[\frac{(n-1)f_1}{(n-1)f_1 + 2f_2}\right]$$

where f_1 is the number of singletons, f_2 is the number of doubletons and n is the number of individuals (Chao and Jost, 2012). We calculated the Hill numbers, or effective number of species, representing species richness (N_0), number of common species (N_1) and number of dominant species (N_2; Hill, 1973). To compare the Hill numbers among vegetation types, we calculated their 95% confidence intervals (Chao and Jost, 2015). These analyses were conducted with the package 'iNEXT' (Hsieh et al., 2016) in R version 1.1.383 (R Core Team, 2017).

2.8.2. Abundance

We compared the abundance among vegetation types (1) for all amphibians, (2) per taxonomic order (Anura including frogs and toads, and Caudata including salamanders), (3) per category of conservation: Non-threatened (including LC and NT categories), Vulnerable, Critically Endangered and Not Included, and (4) habit type (terrestrial, shrub/herbaceous, arboreal and semiaquatic). We used generalized linear models (GLM) with Poisson distribution and log link function, and post hoc tests of contrasts. To consider the spatial arrangement of the plots, distance between plots (Distplot) and distance between vegetation types (Distveg) were added to the models as covariates. We calculated Distplot as the distance between plot_n and the closest plot, and Distveg as the distance between plot_n and the closest edge with a different studied vegetation type. The number of individuals in each plot was obtained by adding the records of the three samplings. These analyses were conducted with the package 'gmodels' (Warnes et al., 2018) in R version 1.1.383 (R Core Team, 2017).

2.8.3. Structure and evenness of the assemblages

To compare the structure and evenness of the assemblages among vegetation types, we used rank abundance curves and calculated the Hill's evenness index, using the formula $E_{1:0} = N_1 / N_0$. Where N_1 is the number of common species and N_0 is the species richness (Hill, 1973).

2.8.4. Species composition and assemblage structure

To compare species composition among vegetation types, we built one dendrogram using the Jaccard similarity index (presence-absence matrix), and another using the Bray-Curtis similarity index (abundance matrix). For the latter dendrogram, to reduce the influence of the most abundant species, we used the chord transformation (Legendre and Legendre, 2012). To check statistical differences between vegetation types, we conducted a permutational multivariate analysis of variance (Permanova; 999 permutations) for both Jaccard and Bray-Curtis indexes, considering Distplot and Distveg as covariates. These analyses were conducted with the package 'vegan' (Oksanen et al., 2016) in R version 1.1.383 (R Core Team, 2017).

2.8.5. Relationship between amphibian recovery and landscape and habitat variables

Prior to construction of the models to determine the variables related to the recovery of species richness and abundance, we selected predictor variables based on Pearson correlation tests among all of the landscape and habitat variables. The variables selected to construct the models were: distance to the stream, distance to the cloud forest, tree density, canopy cover, leaf litter cover, bare soil cover, non-grass herbaceous plant cover, the fern *P. arachnoideum* cover, shrub cover, epiphyte cover and number of fallen trunks (Appendix A). We eliminated highly and significantly correlated variables ($R \geq 0.6$ $p < 0.05$): tree height, basal area and exotic grass cover (Appendix B).

The selected landscape and habitat variables were standardized with the scale function and included in a GLM with Poisson distribution and log link function to determine their effect on amphibian species richness and abundance. We included the 11 variables selected, seeking the best set of predictor variables using a backwards stepwise process. We selected the best model using the second order Akaike Information Criterion (AICc) and defining a value $\Delta\text{AICc} \leq 2$. The models were constructed using the packages 'gmodels' (Warnes et al., 2018), 'MASS' (Venables and Ripley, 2002) and 'geiger' (Harmon et al., 2008) in R version 1.1.383 (R Core Team, 2017).

3. Results

3.1. Species richness, number of common species and number of dominant species

We found a total of 822 individuals of 13 species (9 anurans and 4 salamanders). Five species were considered threatened (three in the Vulnerable and two in the Critically Endangered IUCN categories), seven were considered not threatened (five in the Least Concern and two in the Near Threatened categories) and one species was not included in the IUCN Red List. The two Critically Endangered species were found in the cloud forest and in the three forests under restoration, but not in the cattle pasture (Table 1).

Sample coverage in all vegetation types was > 98%. Species richness was significantly higher in the cloud forest, A23 and P13 than in the cattle pasture. Species richness in P23 was similar to that of all of the other vegetation types (Fig. 2). The numbers of common species and of dominant species were significantly higher in the cloud forest than in the other vegetation types, among which no significant differences were found (Fig. 2). In terms of taxonomic order, anuran species richness was similar among all vegetation types, while salamander species richness was lower in the cattle pasture than in the other vegetation types (Table 1).

Table 1
Species of amphibians recorded in five vegetation types, their abundance, conservation status and habit in a landscape of the mountainous region of central Veracruz, Mexico. CP = cattle pasture, P13 = 13-year-old forest under passive restoration, P23 = 23-year-old forest under passive restoration, A23 = 23-year-old forest under active restoration and CF = cloud forest. Conservation status categories according to the IUCN Red List: LC = Least Concern, NT = Near Threatened, VU = Vulnerable and CR = Critically Endangered. Habit types: S = semiaquatic, T = terrestrial, H = shrub-herbaceous plant and A = arboreal.

ID	Species	Vegetation types					IUCN	Habit
		CP	P13	P23	A23	CF		
	Anura							
	Bufonidae							
Iv	*Incilius valliceps*	1					LC	T
	Craugastoridae							
Cm	*Craugastor mexicanus*		3	10	12	13	LC	T
Cp	*Craugastor pygmaeus*	8	5	16	19	14	VU	T
Cr	*Craugastor rhodopis*	9	56	88	113	57	VU	T
	Eleutherodactylidae							
Ec	*Eleutherodactylus cystignathoides*	4	3	3	2	1	LC	T
	Centrolenidae							
Hf	*Hyalinobatrachium fleischmanni*		6	5	12	16	LC	A
	Hylidae							
Ct	*Charadrahyla taeniopus*		3	1	4	29	VU	A
Rm	*Rheohyla miotympanum*	41	9	26	38	29	NT	H
	Ranidae							
Lb	*Lithobates berlandieri*	8					LC	S
	Caudata							
	Plethodontidae							
Ac	*Aquiloeurycea cafetalera*		5	5	7	20		T
Bp	*Bolitoglossa platydactyla*	1					NT	T
Pt	*Parvimolge townsendi*		17	10	28	53	CR	T
Tp	*Thorius pennatulus*		2		3	7	CR	T
	Species richness	7	10	9	10	10		
	Abundance	72	109	164	238	239		

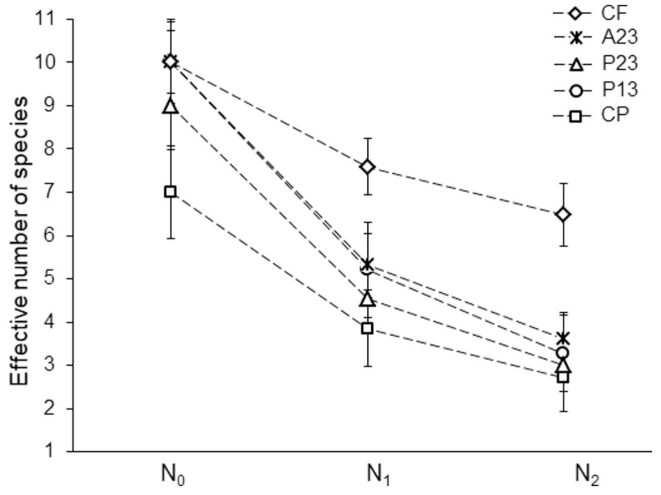

Fig. 2. Species richness of amphibians (N_0), number of common species (N_1) and number of dominant species (N_2) in five vegetation types studied in the mountainous region of central Veracruz, Mexico. CP = cattle pasture, P13 = 13-year-old forest under passive restoration, P23 = 23-year-old forest under passive restoration, A23 = 23-year-old forest under active restoration and CF = cloud forest. Error bars denote 95% confidence intervals.

3.2. Abundance

Abundance of all amphibians was significantly higher in the cloud forest and in A23 than in the other vegetation types. Anuran abundance was higher in A23, while that of salamanders was higher in the cloud forest than in the other vegetation types (Table 1; Fig. 3a–b; Appendix C).

Regarding conservation status, the abundance of Critically Endangered amphibians was higher in the cloud forest, while the abundance of Vulnerable amphibians was higher in A23 than in the other vegetation types. The lowest abundance of Non-threatened amphibians was recorded in P13 and the highest abundance of amphibians Not Included on the Red List was detected in the cloud forest (Fig. 3c; Appendix C).

Regarding habit type, the abundance of arboreal amphibians was higher in the cloud forest than in the other vegetation types. Abundance of terrestrial amphibians was higher in the cloud forest and in A23. The lowest abundance of amphibians that inhabit the shrub and herbaceous plant stratum was recorded in P13. Cattle pasture was the only vegetation type in which we detected individuals of semiaquatic habit (Fig. 3d; Appendix C).

Only the abundance of salamanders, critically endangered species and amphibians that inhabit shrub-herbaceous plants had a significant and negative relationship with distance between vegetation types. The distance between plots had no significant effect on abundance patterns between vegetation types (Appendix C).

3.3. Structure and evenness of the assemblages

The highest evenness value was recorded in the cloud forest, coinciding with the highest number of common species recorded in this vegetation type. In the cloud forest, 8 of the 10 species present were common species. In the other vegetation types, around half of the species were common species (Fig. 4). The common amphibians of the cloud forest were mainly threatened forest specialist species, including two salamander species (*Parvimolge townsendi* and *Thorius pennatulus*) that were present at a lower abundance in the three forests under restoration, but not recorded at all in the cattle pasture. The cloud forest was the only vegetation type where a co-dominance of two species was observed, both of which were terrestrial and threatened: the frog *Craugastor rhodopis* and the salamander *P. townsendi*. In the forests under restoration, the dominant species was the threatened forest specialist frog *C. rhodopis*. Other forest specialists, such as *Charadrahyla taeniopus* and *Aquiloeurycea cafetalera*, were recorded in the forests under restoration, but their abundances were lower than in the cloud forest. In the cattle pasture, the dominant species was the non-threatened frog *Rheohyla miotympanum* (Fig. 4).

3.4. Species composition and assemblage structure

Considering the presence-absence data, the PERMANOVA indicated significant differences in species composition between vegetation types ($F = 2.7$; $p = 0.001$), with no effect of distance between plots ($F = 0.5$; $p = 0.7$) or distance between vegetation types ($F = 1.1$; $p = 0.3$). The similarity of species composition of the cloud forest was 100% with A23 and P13, and 90% with P23. These four vegetation types only shared 32% of species with the cattle pasture (Fig. 5a).

Based on the abundance data, the PERMANOVA indicated significant differences in assemblage structure and species composition between vegetation types ($F = 2.5$; $p = 0.01$), with no effect of distance between plots ($F = 1.1$; $p = 0.3$) or distance between vegetation types ($F = 0.7$; $p = 0.5$). The three forests under restoration had only 38% similarity with the cloud forest, while the four forests had only 5% similarity with the cattle pasture. Based on abundance, the highest similarity values were observed among the forests under restoration, ranging from 58 to 61% (Fig. 5b).

3.5. Relationship between amphibian recovery and landscape and habitat variables

Total amphibian species richness and anuran species richness rose

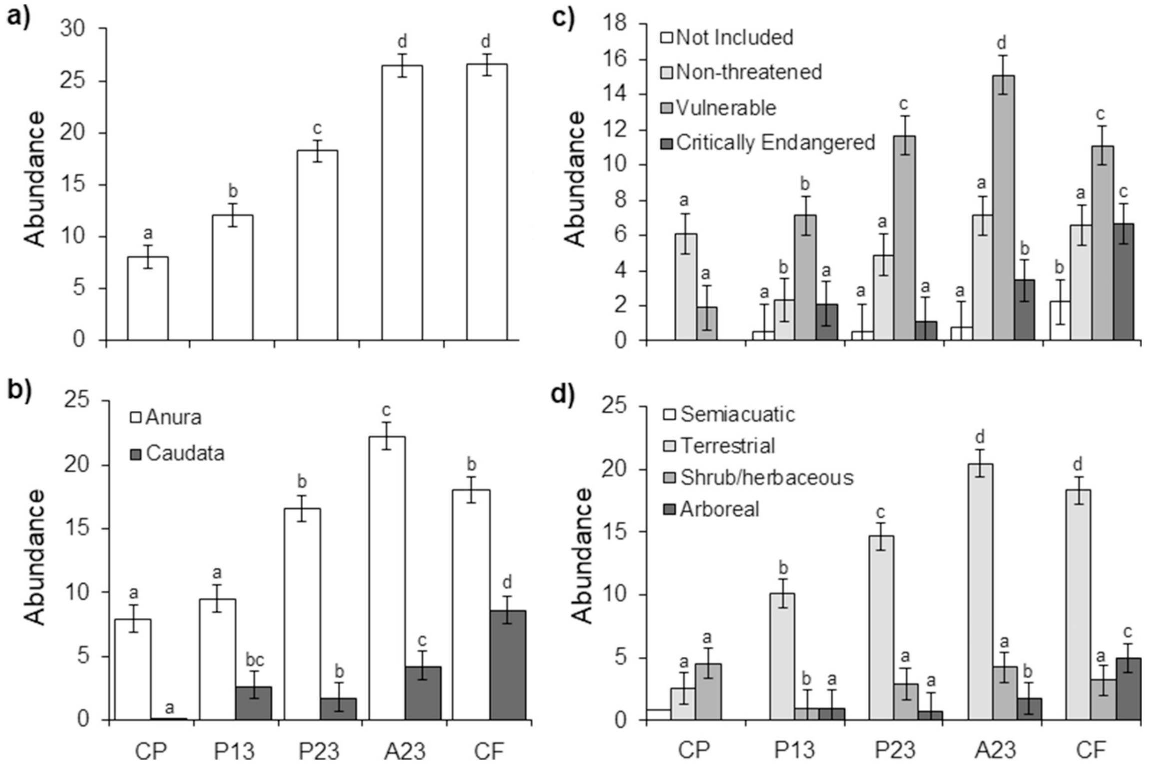

Fig. 3. (a) Abundance of all amphibians, (b) abundance per taxonomic order, (c) abundance per risk category of the IUCN Red List, and (d) abundance per habit type of the species recorded in five vegetation types studied in the mountainous region of central Veracruz, Mexico. CP = cattle pasture, P13 = 13-year-old forest under passive restoration, P23 = 23-year-old forest under passive restoration, A23 = 23-year-old forest under active restoration and CF = cloud forest. Each graph presents the mean and standard error values. Different letters above the bars denote significant differences ($p < 0.001$) according to generalized linear models and post hoc tests of contrasts (Appendix C).

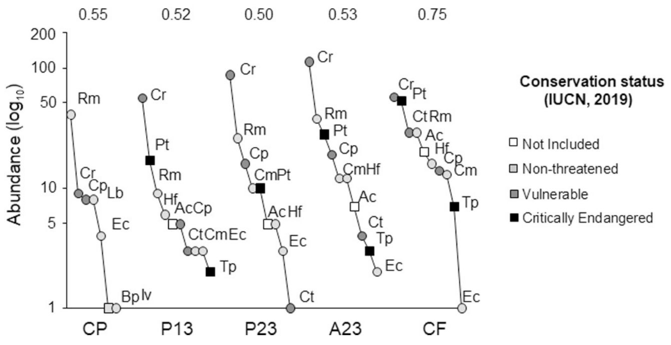

Fig. 4. Rank abundance curves of the amphibian assemblages recorded in five vegetation types studied in the mountainous region of central Veracruz, Mexico. CP = cattle pasture, P13 = 13-year-old forest under passive restoration, P23 = 23-year-old forest under passive restoration, A23 = 23-year-old forest under active restoration and CF = cloud forest. The Hill's evenness index ($E_{1:0}$) is presented above the rank abundance curves for each vegetation type. Circles indicate species of the order Anura and squares indicate species of the order Caudata. The codes for each species are provided in Table 1.

with increasing canopy cover and proximity to the stream. Salamander species richness increased with greater leaf litter and canopy cover. Abundance of all of the amphibians was positively related to leaf litter and non-grass herbaceous plant cover, as well as to proximity to the stream. Anuran abundance increased with greater leaf litter, non-grass herbaceous plant and epiphyte cover, proximity to the stream and proximity to the cloud forest. Salamander abundance increased with greater leaf litter, non-grass herbaceous plant and canopy cover, and with proximity to the stream (Appendix D).

4. Discussion

Our results show that strategies of both passive and active cloud forest restoration implemented close to a remnant of conserved forest generate secondary forests that serve as refuge for the amphibians, including threatened species. Amphibian species richness and species composition, without considering abundance, can recover under both restoration strategies, even after only 13 years of forest recovery with moderate disturbance. Amphibian abundance only reaches values similar to those recorded in the forest in sites that have undergone active restoration for 23 years. However, for Critically Endangered species, none of the restoration strategies yielded abundances similar to those found in the mature cloud forest. Our results also suggest that during cloud forest restoration, terrestrial anuran abundance recovers more quickly than that of arboreal anurans and plethodontid salamanders. This suggests that the amphibians respond differentially to the processes of forest restoration, depending on morphological and physiological traits associated with the taxonomic group, as well as on the habit of the species. Ernst et al. (2006) suggest that ground-dwelling frogs recovered more rapidly than frogs that rely on water, in a rainforest under passive restoration in Africa. Díaz-García et al. (2017) found in Mexico that three groups of terrestrial amphibians present increased abundance in a cloud forest under active restoration: (1) ground-dwelling frogs with pulmonary respiration, (2) toads with pulmonary respiration and aquatic larvae, and (3) ground-dwelling salamanders with cutaneous respiration.

4.1. Recovery of amphibian diversity in cloud forest restoration

The patterns of recovery of amphibian diversity differ depending on the particular metric or community attribute under consideration. In our study, species richness was the first community attribute to recover,

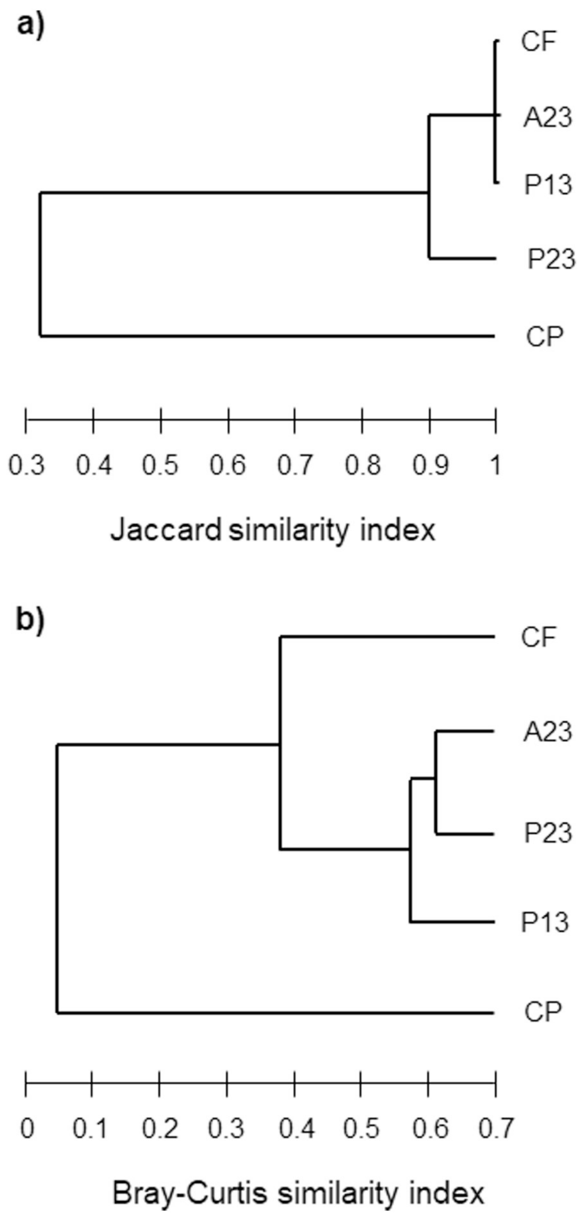

Fig. 5. Dendrograms of similarity in the amphibian species composition in five vegetation types studied in the mountainous region of central Veracruz, Mexico. CP = cattle pasture, P13 = 13-year-old forest under passive restoration, P23 = 23-year-old forest under passive restoration, A23 = 23-year-old forest under active restoration and CF = cloud forest. a) Produced from presence-absence data using the Jaccard index, and b) produced from abundance data using the Bray-Curtis index. In both dendrograms: 0 = completely different and 1 = completely similar.

even under a relatively recent process of passive restoration. This pattern has been reported in other evaluations of amphibian recovery under passive restoration processes in Neotropical forests (Urbina-Cardona and Londoño, 2003; Herrera-Montes and Brokaw, 2010; Hilje and Aide, 2012; Cortés-Gómez et al., 2013), and processes of passive (Basham et al., 2016) and active restoration (Díaz-García et al., 2017) adjacent to a cloud forest. For example, forests under 10–12 years of passive restoration in Costa Rica and Colombia and forests under 7 years of active restoration in Mexico have amphibian species richness similar to that of the reference forest (Hilje and Aide, 2012; Cortés-Gómez et al., 2013; Díaz-García et al., 2017). However, other attributes such as abundance and species composition in forests under passive or active restoration do not reach similar levels to those in the reference forest (Díaz-García et al., 2017; Thompson and Donnelly, 2018). These two community attributes offer important insights into the process of fauna recovery in sites under restoration, since they reflect the availability of the quantity and variety of resources necessary to sustain amphibian populations (Dunn, 2004; Catterall, 2018), particularly those of the forest specialist species.

After 23 years of active forest recovery, the characteristics at habitat level necessary for recovery of amphibian abundance, such as a closed canopy and high leaf litter, herbaceous plant and epiphyte cover, are reestablished to a greater extent than in the forests under passive restoration (Trujillo-Miranda et al., 2018, see Appendix A). The reestablishment of a closed canopy and the high leaf litter cover reduce temperature variation and maintain high values of ambient humidity (Guariguata and Ostertag, 2001), which are microclimatic conditions required by tropical forest amphibians (Díaz-García et al., 2017; Thompson and Donnelly, 2018). However, since there was no forest under recent active restoration in the studied landscape, we cannot be certain if the recovery of vegetation structure and abundance of amphibians occur more rapidly in active restoration areas. In our study, forests under passive restoration for 13 and 23 years reached 45 and 68%, respectively, of the amphibian abundance present in the cloud forest. If this ecological trajectory were to continue, it could be expected that amphibian abundance in these forests would be similar to that of the cloud forest within a period of 30 to 33 years, coinciding with that reported by Basham et al. (2016) in a cloud forest under passive restoration in Colombia, as well as the time estimated by Hernández-Ordoñez et al. (2015) in a rainforest under passive restoration in Mexico.

In the Neotropical region, forests under restoration for ten years after abandonment of cattle pastures and with an adjacent forest remnant, have been found to share amphibian species with the reference forest (Hilje and Aide, 2012; Basham et al., 2016), which coincides with our results. However, when considering amphibian abundance, the species composition of the three forests under restoration only has 38% similarity with the cloud forest. This occurs because most of the forest specialist species, such as *P. townsendi*, *A. cafetalera*, *T. pennatulus*, *Hyalinobatrachium fleischmanni* and *C. taeniopus*, are less abundant in these forests than in the cloud forest. Forest specialist amphibians exhibit a more limited capacity for dispersal (Catterall, 2018) and tolerate a reduced range of habitat conditions (Ríos-López and Aide, 2007; Klaus and Noss, 2016), which can delay their recovery in restoration areas.

The different responses to cloud forest restoration presented by anurans and salamanders, and by species with different habit type seem mainly to be related to morphological and physiological traits, and to the degree of habitat specialization in each species. For example, the plethodontid salamanders found in the cloud forest seemed to be less resilient to forest disturbance than the anurans. This could be due to the fact that plethodontid salamanders do not have lungs but respire through their skin, for which they require strictly humid and cool environments (Wake and Lynch, 1976). In tropical forests, these microclimatic conditions are only present in the most advanced stages of succession (Guariguata and Ostertag, 2001). Moreover, these salamanders require specific microhabitats such as fallen trunks and epiphytic plants (Sandoval-Comte et al., 2012), which were less common in the forests under restoration and cattle pastures (see Appendix A). Furthermore, salamander abundance decreased with distance between vegetation types. This is probably related to their niche conservatism and low vagility (Welsh and Hodgson, 2013), which can limit their capacity to move from the forest and colonize other vegetation types. To ensure and maximize the recovery of these threatened salamanders, restoration practitioners should select sites close to mature forest and implement strategies complementary to forest restoration, such as the introduction of tree trunks or translocation of epiphytic plants (Fernandez-Barrancos et al., 2017). It is necessary, however, to design studies that evaluate whether such complementary strategies can

contribute significantly to the recovery of these amphibians.

The species that most benefited from both restoration strategies was the threatened forest specialist frog *C. rhodopis*, the abundance of which increased 6–12 times with restoration of the cattle pastures. This could be related to the feeding habits of this frog and to the recovery of potential prey populations. *Craugastor rhodopis* feeds mainly on leaf litter orthopterans, spiders and beetles (Luría-Manzano et al., 2019), arthropods that increase in number with cloud forest restoration (de la Rosa and Negrete-Yankelevich, 2012). On the other hand, recovery of the cloud forest tree frogs *C. taeniopus* and *H. fleischmanni* was lower compared to that of the terrestrial frogs. These tree frogs of the families Hylidae and Centrolenidae are associated with the physical vertical structure of the vegetation, due to the fact that they use the trees to travel, perch and reproduce. Some also deposit their eggs on the leaves (Wells, 2007).

Our results support the idea that proximity to water bodies is a landscape element that determines the persistence and recovery of amphibian diversity in the forest under restoration. Even under recent passive restoration processes, the recovery of threatened amphibian species is facilitated by the presence of streams. Water availability influences vegetation growth (Naiman et al., 2005) and thus the recovery of the microhabitats and microclimatic conditions necessary for forest specialist amphibians (Ficetola et al., 2008). Other landscape factors that determine amphibian recolonization in restoration areas, but were not considered in our study, include the quantity of surrounding forest (Catterall, 2018), complexity of the matrix and connectivity with forest fragments (Thompson and Donnelly, 2018).

Our study region lacks large and continuous mature forest fragments; instead, there is a mosaic of secondary and relatively preserved forest patches interspersed with agricultural and urban land uses (CONABIO, 2010). The reference ecosystem in this study only presents an area of 30 ha and, given its form, contains a large proportion of edge that may have negative edge effects on amphibian diversity (Santos-Barrera and Urbina-Cardona, 2011). However, the mature fragment close to the restoration areas presents a unique opportunity to compare the success of different restoration strategies with high ecological fidelity. Our reference forest presented a total richness of 10 species, which is comparable to the amphibian richness found in six cloud forest remnants that have been studied in the same region (see Murrieta-Galindo et al., 2013; Meza-Parral and Pineda, 2015). These fragments are of between 10 and 120 ha in area and host a total of 16 amphibian species (between 6 and 11 species per fragment, with the largest fragment hosting 9 species), including three threatened forest specialist species that were not recorded in our study. Likewise, in the study region, high indices of amphibian beta diversity have been reported among the cloud forest fragments (Pineda and Halffter, 2004; Meza-Parral and Pineda, 2015). Beta diversity is increased in highly fragmented landscapes in which the degradation or loss of a small forest remnant can have a large impact on total species richness (Fahrig et al., 2019). For this reason, the development of restoration strategies that promote connectivity among forest fragments could increase the value of the forests under restoration in terms of amphibian conservation at the landscape level.

4.2. Implications for the conservation of threatened species and cloud forest restoration practices

There is no general consensus to determine which cloud forest restoration strategy is more efficient in terms of recovering forest cover and mitigating or reversing biodiversity loss. For vegetation, it has been reported that species diversity and structure can be similar to those of mature cloud forest after 40 years of passive restoration (Muñiz-Castro et al., 2006), or seven years of active restoration (Díaz-García et al., 2017). Recently, Trujillo-Miranda et al. (2018) compared different cloud forest restoration strategies in our study region and found that, after 21 years, the recovery of vegetation structure was higher in sites under active restoration compared to those under passive restoration.

Our study is the first to compare amphibian recovery under different forest restoration strategies, and demonstrates that both passive and active cloud forest restoration strategies are of value for amphibian recovery, and thus complement other conservation strategies for highly threatened groups, such as the protection of mature forests. However, given the continued destruction of tropical forests that has led to the reduction of amphibian species and populations (IUCN, 2019; WWF, 2018), there is an urgent need to implement programs of active restoration with mixed plantations of native species because this is more efficient than passive restoration in terms of recovering forest specialist amphibian populations, including species in danger of extinction. It is also recommended that restoration projects should be implemented in sites close to water bodies, and should include practices that accelerate the recovery of populations of threatened fauna, such as the establishment of clusters of stones and logs, or the creation of permanent ponds (Urbina-Cardona et al., 2015).

Restoration projects are commonly implemented on a site-specific basis with no replication, as in our study. The lack of replication therefore limits the inferences to other restoration projects that can be drawn from the results. Ecological restoration would benefit from restoration projects that consider: (1) true replications to assess the recovery of fauna diversity, (2) restoration treatments (either active or passive) of different ages in order to delineate the recovery trajectory, and (3) implementation of restoration treatments at different distances from several reference ecosystems and consideration of larger forest fragments and continuous forest in order to avoid potential edge effects.

It is necessary to continue studying the recovery of fauna under different restoration processes and to include other aspects of diversity, such as functional and phylogenetic diversity. This would allow us to determine more efficiently whether forest restoration is also capable of recovering biodiversity and maintaining the biotic interactions and ecological functions necessary for the resilience of biotic communities to factors of global change.

Funding

This study was funded by the fundamental science project “Atributos funcionales de especies arbóreas y los escenarios para la restauración del paisaje de bosque mesófilo de montaña en el Centro de Veracruz” of the Secretaría de Educación Pública-Consejo Nacional de Ciencia y Tecnología (SEP-CONACYT), numbers CB2014-238831-B. JMDG was the recipient of a postgraduate studies grant (No. 486768) from CONACYT.

CRediT authorship contribution statement

J.M. Díaz-García: Conceptualization, Formal analysis, Investigation, Methodology, Writing - review & editing. **F. López-Barrera:** Conceptualization, Formal analysis, Funding acquisition, Investigation, Methodology, Writing - review & editing. **T. Toledo-Aceves:** Funding acquisition, Formal analysis, Methodology, Writing - review & editing. **E. Andresen:** Formal analysis, Methodology, Writing - review & editing. **E. Pineda:** Conceptualization, Formal analysis, Investigation, Methodology, Writing - review & editing.

Declaration of competing interest

The authors declare that they have no known competing financial interests or personal relationships that could have appeared to influence the work reported in this paper.

Acknowledgements

The cooperative Las Cañadas provided hospitality and other facilities during the fieldwork. Ricardo Romero, Tania de Alba, Estefanía

Oliveras, Raúl Bandín, Juan Acosta, Adrián Acosta and Carlos Nolasco granted permission to work on their properties. Antonio Reyes and Octavio Suárez were our field guides. Sarai Gómez, Adriana Sandoval, María de Jesús Peralta, Juan Fernando Escobar, María Chanel Juárez, Judith Ramos, Paulina García, Arístides García, Enrique Silva, Alfonso Kelly and Víctor Vásquez helped with the fieldwork. Adriana Sandoval, Policarpo Ronzón and Víctor M. Vásquez provided technical and logistic assistance. Adriana Sandoval produced the map of the study area. Keith MacMillan translated this manuscript. Two anonymous reviewers made valuable comments that improved this manuscript. This article was written to comply with the requirements of the Programa de Estudios del Posgrado of the Instituto de Ecología A.C. for the Doctorate of JMDG.

Appendix A. Characteristics of the landscape and habitat present in the five vegetation types studied: CP = cattle pasture, P13 = 13-year-old forest under passive restoration, P23 = 23-year-old forest under passive restoration, A23 = 23-year-old forest under active restoration and CF = cloud forest. Mean and standard deviation (in parentheses) values are presented for all variables

	Vegetation types				
	CP	P13	P23	A23	CF
Landscape variables					
Distance to stream (m)	182.5 (131)	221 (102)	87.3 (58.7)	78.3 (55.2)	60 (25.8)
Distance to CF (m)	1800.6 (205)	501 (137)	1152 (298)	995.1 (322)	0 (0)
Habitat variables					
Tree density (trees/ha)	16.6 (35.5)	289 (173)	383 (141.2)	461.1(89.4)	588.9 (193)
Canopy cover (%)	13.1 (15.3)	67.5 (8.2)	72.3 (5)	76.1 (6)	77.4 (3.7)
Leaf litter cover (%)	7.7 (8.8)	23.1 (15.4)	44.2 (20.8)	50.3 (24.2)	67.8 (13.6)
Bare soil cover (%)	4.7 (10)	7.1 (10.3)	6 (5.7)	2.7 (2.6)	12 (10)
Shrub cover (%)	1 (2.5)	22.1 (11.6)	20.3 (11.2)	15.1 (6.8)	30.8 (13.1)
Cover of non-grass herbaceous plants (%)	2.8 (6.8)	48.8 (16.6)	34 (22)	35.4 (26.6)	16.6 (6.4)
Cover of *Pteridium arachnoideum* (%)	0 (0)	9.3 (16.1)	5 (9.9)	1 (1)	1 (1)
Epiphyte cover (%)	7.3 (11.4)	7.5 (8.3)	9.5 (12.2)	11.1 (8.7)	49 (25.5)
Number of fallen trunks	0.7 (1.25)	1 (0.6)	2.1(1.5)	3.2 (1.8)	5.6 (1.6)

Appendix B. Matrix with Pearson's correlation coefficients (upper triangle) and p-values (lower triangle) for landscape and habitat variable correlations. DS = distance to stream, DCF = distance to cloud forest, TD = tree density, TH = tree height, BA = basal area, CC = canopy cover, LLC = leaf litter cover, BSC = bare soil cover, EGC = exotic grass cover, CNHG = Cover of non-grass herbaceous plants, CPT = Cover of *Pteridium arachnoideum*, SC = shrub cover, EC = epiphyte cover and FT = number of fallen trunks

	DS	DCF	TD	TH	BA	CC	LLC	BSC	EGC	CNGH	CPT	SC	EC	FT
DS	1	0.15	−0.34	−0.50	−0.45	−0.45	−0.50	−0.24	0.45	0.04	−0.01	−0.04	−0.43	−0.46
DCF	0.33	1	−0.51	−0.52	−0.60	−0.60	−0.45	−0.25	0.70	−0.36	−0.14	−0.51	−0.48	−0.50
TD	0.02	0.01	1	0.80	0.70	0.71	0.60	0.38	−0.73	0.21	−0.05	0.55	0.43	0.63
TH	0.05	0.01	0.01	1	0.75	0.81	0.55	0.27	−0.66	0.18	−0.01	0.53	0.59	0.60
BA	0.05	0.09	0.01	0.01	1	0.70	0.65	0.24	−0.64	−0.07	−0.17	0.50	0.66	0.70
CC	0.05	0.05	0.08	0.05	0.01	1	0.60	0.19	−0.94	0.50	0.12	0.61	0.29	0.50
LLC	0.01	0.01	0.01	0.01	0.01	0.01	1	0.09	−0.67	−0.26	−0.02	0.36	0.45	0.59
BSC	0.11	0.09	0.01	0.07	0.11	0.21	0.57	1	−0.33	0.00	0.02	0.27	0.33	0.26
EGC	0.01	0.01	0.01	0.01	0.01	0.01	0.01	0.03	1	−0.50	−0.09	−0.61	−0.33	−0.60
CNGH	0.77	0.02	0.16	0.25	0.67	0.01	0.09	0.98	0.01	1	0.14	0.34	−0.18	−0.09
CPT	0.96	0.34	0.76	0.97	0.27	0.44	0.90	0.88	0.55	0.35	1	−0.03	−0.05	−0.24
SC	0.81	0.01	0.06	0.01	0.01	0.01	0.01	0.07	0.01	0.02	0.84	1	0.32	0.48
EC	0.5	0.05	0.1	0.01	0.01	0.05	0.01	0.06	0.03	0.24	0.76	0.03	1	0.56
FT	0.6	0.05	0.07	0.01	0.01	0.01	0.05	0.08	0.01	0.55	0.11	0.01	0.01	1

Appendix C. Results of the generalized linear models (n = 45), used to compare the abundance of all amphibians, per taxonomic order, per conservation status category and per vegetation type (Vegtype) and considering the distance between plots (Distplot) and the distance between vegetation types (Distveg) as covariates. Cattle pasture was the only vegetation type in which we detected individuals of semiaquatic habit

			Deviance	Degrees of freedom	p value
All amphibians		Vegtype	145.7	40	0.001
		Distplot	1.31	39	0.2
		Distveg	9.8	38	0.06
Taxonomic order	Anura	Vegtype	91.7	40	0.001
		Distplot	4.5	39	0.03
		Distveg	0.3	38	0.8
	Caudata	Vegtype	114.7	40	0.001
		Distplot	3.26	39	0.07
		Distveg	6.9	38	0.001

Conservation status	Not Included	Vegtype	31.1	40	0.001
		Distplot	2.4	39	0.12
		Distveg	1.8	38	0.17
	Non-threatened	Vegtype	27.7	40	0.001
		Distplot	4.3	39	0.03
		Distveg	2.3	38	0.07
	Vulnerable	Vegtype	119.7	40	0.001
		Distplot	0.9	39	0.32
		Distveg	4.3	38	0.03
	Critically Endangered	Vegtype	99.4	40	0.001
		Distplot	7.1	39	0.07
		Distveg	12.5	38	0.001
Habit types	Terrestrial	Vegtype	171.1	40	0.001
		Distplot	0.01	39	0.9
		Distveg	0.09	38	0.7
	Shrub-herbaceous plant	Vegtype	46.1	40	0.001
		Distplot	4.1	39	0.07
		Distveg	5.1	38	0.04
	Arboreal	Vegtype	78.7	40	0.001
		Distplot	3.7	39	0.06
		Distveg	2.9	38	0.08
	Semiaquatic				

Appendix D. Results of the generalized linear models selected to explain the recovery of the richness and abundance of amphibians recorded in the five vegetation types studied

	Estimate	Std. error	z value	p value
Species richness				
All amphibians (Deviance = 70.92, AIC = 198.86, ΔAICc < 2)				
Canopy cover	0.33	0.11	2.93	< 0.01
Distance to stream	−0.21	0.10	−2.02	< 0.05
Epiphyte cover	0.11	0.06	1.68	0.09
Anura (Deviance = 49.71, AIC = 178.55, ΔAICc < 2)				
Canopy cover	0.25	0.11	2.16	< 0.05
Distance to stream	−0.24	0.11	−2.17	< 0.05
Caudata (Deviance = 55.11, AIC = 100.94, ΔAICc < 2)				
Leaf litter cover	0.45	0.18	2.44	< 0.01
Canopy cover	0.91	0.45	2.02	< 0.05
Abundance				
All amphibians (Deviance = 472.11, AIC = 350.15, ΔAICc < 2)				
Distance to stream	−0.48	0.06	−7.55	< 0.001
Leaf litter cover	0.43	0.06	6.67	< 0.001
Cover of non-grass herbaceous plants	0.37	0.06	6.19	< 0.001
Distance to CF	0.11	0.05	2.11	0.06
Bare soil cover	0.07	0.04	1.86	0.06
Epiphyte cover	0.07	0.04	1.64	0.10
Anura (Deviance = 360.34, AIC = 351.32, ΔAICc < 2)				
Distance to stream	−0.41	0.06	−6.27	< 0.001
Cover of non-grass herbaceous plants	0.43	0.06	7.30	< 0.001
Leaf litter cover	0.35	0.06	5.48	< 0.001
Distance to CF	0.28	0.06	4.63	< 0.001
Epiphyte cover	0.19	0.04	4.05	< 0.001
Caudata (Deviance = 272.32, AIC = 168.25, ΔAICc < 2)				
Leaf litter cover	1.36	0.34	3.92	< 0.001
Bare soil cover	0.56	0.12	4.45	< 0.001
Canopy cover	1.42	0.51	2.77	< 0.01
Distance to stream	−0.65	0.23	−2.80	< 0.01
Epiphyte cover	−0.35	0.19	−3.26	< 0.01
Cover of non-grass herbaceous plants	0.64	0.31	2.04	< 0.05
Distance to CF	−0.27	0.15	−1.73	0.08

References

Aide, T.M., Ruiz-Jaen, M.C., Grau, H.R., 2010. What is the state of tropical montane cloud forest restoration? In: Bruijnzeel, L.A., Scatena, F.N., Hamilton, L.S. (Eds.), Tropical Montane Cloud Forests Science for Conservation and Management. Cambridge University Press, Cambridge, UK, pp. 101–110. https://doi.org/10.1017/CBO9780511778384.010.

Álvarez-Aquino, C., Williams-Linera, G., Newton, A.C., 2004. Experimental native tree seedling establishment for the restoration of a Mexican Cloud Forest. Restor. Ecol. 12, 412–418. https://doi.org/10.1111/j.1061-2971.2004.00398.x.

Ash, A.N., 1997. Disappearance and return of Plethodontid salamanders to clearcut plots in the southern Blue Ridge Mountains. Conserv. Biol. 11, 983–989. https://doi.org/10.1046/j.1523-1739.1997.96172.x.

Basham, E.W., González del Pliego, P., Acosta-Galvis, A.R., Woodcock, P., Medina-Uribe, C.A., Haugaasen, T., Gilroy, J.J., Edwards, D.P., 2016. Quantifying carbon and amphibian co-benefits from secondary forest regeneration in the Tropical Andes. Anim. Conserv. 19, 548–560. https://doi.org/10.1111/acv.12276.

Bishop, P.J., Angulo, A., Lewis, J.P., Moore, R.D., Rabb, G.B., Garcia-Moreno, J., 2012. The amphibian extinction crisis – what will it take to put the action into the amphibian conservation action plan? In: S.A.P.I.E.N.S. vol. 5. pp. 97–111.

Catterall, C.P., 2018. Fauna as passengers and drivers in vegetation restoration: a synthesis of processes and evidence. Ecol. Manag. Restor. 19, 54–62. https://doi.org/10.1111/emr.12306.

Challenger, A., 1998. Utilización y conservación de los ecosistemas terrestres de México: pasado, presente y futuro. CONABIO-Instituto de Biología UNAM, México.

Chao, A., Jost, L., 2012. Coverage-based rarefaction and extrapolation: standardizing samples by completeness rather than by size. Ecology (12), 2533–2547. https://doi.org/10.1890/11-1952.1.
Chao, A., Gotelli, N.J., Hsieh, T.C., Sander, E.L., Ma, K.H., Colwell, R.K., Ellison, A.M., 2014. Rarefaction and extrapolation with Hill numbers: a framework for sampling and estimation in species diversity studies. Ecol. Monogr. 84, 45–67. https://doi.org/10.1890/13-0133.1.
Chao, A., Jost, L., 2015. Estimating diversity and entropy profiles via discovery rates of new species. Methods Ecol. Evol. 6, 873–882. https://doi.org/10.1111/2041-210X.12349.
Comisión Nacional para el conocimiento y uso de la Biodiversidad, 2010. El Bosque Mesófilo de Montaña en México: Amenazas y Oportunidades para su Conservación y Manejo Sostenible. Comisión Nacional para el Conocimiento y Uso de la Biodiversidad, México.
Cortés-Gómez, A.M., Castro-Herrera, F., Urbina- Cardona, J.N., 2013. Small changes in vegetation structure create great changes in amphibian ensembles in the Colombian Pacific rainforest. Trop. Conserv. Sci. 6, 749–769. https://doi.org/10.1177/194008291300600604.
Crouzeilles, R., Ferreira, M.S., Chazdon, R.L., Lindenmayer, D.B., Sansevero, J.B.B., Monteiro, L., Iribarrem, A., Latawiec, A., Strassburg, B.B.N., 2017. Ecoligial restoration success is higher for natural regeneration than for active restoration in tropical forests. Sci. Adv. 3, e1701345. https://doi.org/10.1126/sciadv.1701345.
Crump, M.L., Scott, N.J., 1994. Visual encounter surveys. In: Heyer, W.R., Donnelly, M.A., McDiarmid, R.W., Hayek, L.A.C., Foster, M.S. (Eds.), Measuring and Monitoring Biological Diversity. Standard Methods for Amphibians. Smithsonian Institution Press, Washington, DC, pp. 84–92.
de la Rosa, I.N., Negrete-Yankelevich, S., 2012. Spatial distribution of soil macrofauna in cloud forest, secondary forest and grasslands in La Cortadura reserve, Coatepec, Veracruz, Mexico. Rev. Mex. Biodiv. 83, 201–215.
Díaz-García, J.M., Pineda, E., López-Barrera, F., Moreno, C.E., 2017. Amphibian species and functional diversity as indicators of restoration success in tropical montane forest. Biodivers. Conserv. 26, 2569–2589. https://doi.org/10.1007/s10531-017-1372-2.
Duellman, W.E., Trueb, L., 1986. Biology of Amphibians. Johns Hopkins University Press, Maryland.
Dunn, R.R., 2004. Recovery of faunal communities during tropical forest regeneration. Conserv. Biol. 18, 302–309.
Ernst, R., Linsenmair, K.E., Rödel, M.O., 2006. Diversity erosion beyond the species level: dramatic loss of functional diversity after selective logging in two tropical amphibian communities. Biol. Conserv. 133, 143–155. https://doi.org/10.1016/j.biocon.2006.05.028.
Fahrig, L., et al., 2019. Is habitat fragmentation bad for biodiversity? Biol. Conserv. 230, 179–186. https://doi.org/10.1016/j.biocon.2018.12.026.
Fernandez-Barrancos, E., Leighton Reid, J., Aronson, J., 2017. Tank bromeliad transplants as an enrichment strategy in southern Costa Rica: restoration enrichment using bromeliad transplants. Restor. Ecol. 25, 569–576. https://doi.org/10.1111/rec.12463.
Ficetola, G.F., Furlani, D., Colombo, G., De Bernardi, F., 2008. Assessing the value of secondary forest for amphibians: *Eleutherodactylus* frogs in a gradient of forest alteration. Biodivers. Conserv. 17, 2185–2195. https://doi.org/10.1007/s10531-007-9280-5.
Gascon, C., Collins, J.P., Moore, R.D., Church, D.R., McKay, J.E., Mendelson, J.R., 2007. Amphibian Conservation Action Plan. IUCN/SSC Amphibian Specialist Group, Gland, Switzerland and Cambridge, UK.
Gual-Díaz, M., Mayer-Goyenechea, I., 2014. Anfibios del bosque mesófilo de montaña. In: Gual-Díaz, M., Rendón-Correa, A. (Eds.), Bosques mesófilos de montaña de México: diversidad, ecología y manejo. Comisión Nacional para el Conocimiento y Uso de la Biodiversidad, México.
Guariguata, M.R., Ostertag, R., 2001. Neotropical secondary forest succession: changes in structural and functional characteristics. For. Ecol. Manag. 148, 185–206. https://doi.org/10.1016/S0378-1127(00)00535-1.
Harmon, L.J., Jason, T.W., Chad, D.B., Richard, E.G., Challenger, W., 2008. GEIGER: investigating evolutionary radiations. Bioinformatics 24, 129–131. https://doi.org/10.1093/bioinformatics/btm538.
Heinen, J.T., 1992. Comparisons of the leaf litter herpetofauna in abandoned cacao plantations and primary rain forest in Costa Rica: some implications on fauna restoration. Biotropica 24, 431–439. https://doi.org/10.2307/2388614.
Hernández-Ordoñez, O., Urbina-Cardona, J.N., Martinez-Ramos, M., 2015. Recovery of amphibian and reptile assemblages during old-field succession of tropical rain forests. Biotropica 47, 377–388. https://doi.org/10.1111/btp.12207.
Herrera-Montes, A., Brokaw, N., 2010. Conservation value of tropical secondary forest: a herpetofaunal perspective. Biol. Conserv. 143, 1414–1422. https://doi.org/10.1016/j.biocon.2010.03.016.
Hilje, B., Aide, T.M., 2012. Recovery of amphibian species richness and composition in a chronosequence of secondary forests, northeastern Costa Rica. Biol. Conserv. 146, 170–176. https://doi.org/10.1016/j.biocon.2011.12.007.
Hill, M., 1973. Diversity and evenness: a unifying notation and its consequences. Ecology 54, 427–432.
Homyack, J., Hass, C., Hopkins, W., 2011. Energetics of surface-active terrestrial salamanders in experimentally harvested forest. J. Wildl. Manag. 75, 1267–1278. https://doi.org/10.1002/jwmg.175.
Hsieh, T.C., Ma, K.H., Chao, A., 2016. iNEXT: an R package for rarefaction and extrapolation of species diversity (Hill numbers). Methods Ecol. Evol. 2016, 1–6. https://doi.org/10.1111/2041-210X.12613.
IPBES, 2019. Media release: nature's dangerous decline 'unprecedented'; species extinction rates 'accelerating'. https://www.ipbes.net/news/Media-Release-Global-Assessment, Accessed date: 10 June 2019.
IUCN, 2019. The IUCN Red List of Threatened Species. Version 2019-1. https://www.iucnredlist.org/, Accessed date: 10 June 2019.
Klaus, J.M., Noss, R.F., 2016. Specialist and generalist amphibians respond to wetland restoration treatments. J. Wildl. Manag. 80, 1106–1119. https://doi.org/10.1002/jwmg.21091.
Korhonen, L., Korhonen, K.T., Rautiainen, M., Stenberg, P., 2006. Estimation of forest canopy cover: comparison of field measurement techniques. Silva Fenn 40, 577–588. https://doi.org/10.14214/sf.315.
Legendre, P., Legendre, L., 2012. Numerical Ecology. Elsevier Press, San Diego, USA.
Luría-Manzano, R., Oropeza-Sánchez, M.T., Aguilar-López, J.L., Díaz-García, J.M., Pineda, E., 2019. Dieta de la rana de hojarasca *Craugastor rhodopis* (Anura: Craugastoridae): una especie abundante en la región montañosa del este de México. Rev. Biol. Trop. 67, 196–205. https://doi.org/10.15517/RBT.V67I1.33135.
McDonald, T., Gann, G.D., Jonson, J., Dixon, K.W., 2016. International standards for the practice of ecological restoration including principles and key concepts. Society for Ecological Restoration, Washington, D.C.
Millennium Ecosystem Assessment, 2005. Ecosystems and Human Well-being: Synthesis. Island Press, Washington, DC.
Meza-Parral, Y., Pineda, E., 2015. Amphibian diversity and threatened species in a severely transformed neotropical region in Mexico. PLoS One 10, e0121652. https://doi.org/10.1371/journal.pone.0121652.
Murrieta-Galindo, R., López-Barrera, F., Gónzalez-Romero, A., Parra-Olea, G., 2013. Matriz and habitat quality in a montane cloud-forest landscape: amphibians in coffee plantations in central Veracruz, Mexico. Wildl. Res. 40, 25–35. https://doi.org/10.1071/WR12076.
Muñiz-Castro, M.A., Williams-Linera, G., Benayas, J.M., 2006. Distance effect from cloud forest fragments on plant community structure in abandoned pastures in Veracruz, Mexico. J. Tropical Ecol. 22, 431–440. https://doi.org/10.1017/S0266467406003221.
Naiman, R.J., Decamps, H., McClain, M.E., Likens, G.E., 2005. Riparia: Ecology, Conservation and Management of Streamside Communities. Elsevier Press, San Diego, USA.
Nori, J., Lemes, P., Urbina-Cardona, N., Baldo, D., Lescano, J., Loyola, R., 2015. Amphibian conservation, land-use changes and protected areas: a globar review. Biol. Conserv. 191, 367–374. https://doi.org/10.1016/j.biocon.2015.07.028.
Nowakowski, A.J., Frishkoff, L.O., Thompson, M.E., Smith, T.M., Todd, B.D., 2018. Phylogenetic homogenization of amphibian assemblages in human-altered habitats across the globe. Proc. Natl. Acad. Sci. 115, E3454–E3462. https://doi.org/10.1073/pnas.1714891115.
Oksanen, J., Guillaume-Blanchet, F., et al., 2016. Community Ecology Package "Vegan" for R Version 2.4.
Pineda, E., Halffter, G., 2004. Species diversity and habitat fragmentation: frogs in a tropical montane landscape in Mexico. Biol. Conserv. 117, 499–508. https://doi.org/10.1016/j.biocon.2003.08.009.
Primack, R.B., 2010. Essentials of Conservation Biology. Sinauer Associates Publishers, Sunderland, USA.
Pyron, R.A., 2018. Global amphibian declines have winners and losers. Proc. Natl. Acad. Sci. 115, 3739–3741. https://doi.org/10.1073/pnas.1803477115.
R Core Team, 2017. A Language and Environment for Statistical Computing. https://www.R-project.org/.
Ramírez-Soto, A., Lucio-Palacio, C.R., Rodríguez-Mesa, R., Sheseña-Hernández, I., Farhat, F.N., Villa-Bonilla, B., Landa-Libreros, L., Gutiérrez-Sosa, G., Trujillo-Santos, O., Gómez-Sánchez, I., 2018. Restoration of tropical montane cloud forests: a six-prong strategy. Restor. Ecol. 26, 106–211. https://doi.org/10.1111/rec.12660.
Reid, J.L., Fagan, M.E., Zahawi, R.A., 2018. Positive site selection bias in meta-analyses comparing natural regeneration to active forest restoration. Sci. Adv. 4, eaas9143. https://doi.org/10.1126/sciadv.aas9143.
Rey-Benayas, J.M., Bullock, J.M., Newton, A.C., 2008. Creating woodland islets to reconcile ecological restoration, conservation and agricultural land use. Front. Ecol. Environ. 6, 329–336. https://doi.org/10.1890/070057.
Ríos-López, N., Aide, T.M., 2007. Herpetofaunal dynamics during secondary succession. Herpetologica 63, 35–50. https://doi.org/10.1655/0018-0831(2007)63[35:HDDSS]2.0.CO;2.
Romero-González, R., 2018. Ganadería agroecológica en una zona de bosque de niebla. In: Halffter, G., Cruz, M., Huerta, C. (Eds.), Ganadería sustentable en el Golfo de México. Instituto de Ecología A.C, México, pp. 345–367.
Sandoval-Comte, A., Pineda, E., Aguilar-López, J.L., 2012. In search of critically endangered species: the current situation of two tiny salamanders species in the Neotropical mountains of Mexico. PLoS ONE 7, e34023. https://doi.org/10.1371/journal.pone.0034023.
Santos-Barrera, G., Urbina-Cardona, N., 2011. The role of matrix-edge dynamics of amphibian conservation in tropical montane fragmented landscapes. Rev. Mex. Biodiv. 82, 679–687. https://doi.org/10.22201/ib.20078706e.2011.2.463.
Scatena, F.N., Bruijnzeel, L.A., Bubb, P., Das, S., 2010. Setting the stage. In: Bruijnzeel, L.A., Scatena, F.N., Hamilton, L.S. (Eds.), Tropical Montane Cloud Forests. Science for Conservation and Management. Cambridge University Press, Cambridge, pp. 3–13.
Society for Ecological Restoration, 2004. The SER international primer on ecological restoration. SER. Science and Policy Working Group, Washington, DC.
Stuart, A.N., Chanson, J.S., Cox, N.A., Young, B.E., Rodrigues, A.S.L., Fischman, D.L., Waller, R.W., 2004. Status and trends of amphibian declines and extinctions worldwide. Science 306, 1783–1786. https://doi.org/10.1126/science.1103538.
Thompson, M.E., Donnelly, M.A., 2018. Effects of secondary forest succession on amphibians and reptiles: a review and meta-analysis. Copeia 106, 10–19. https://doi.org/10.1643/CH-17-654.
Toledo-Aceves, T., Meave, J.A., González-Espinoza, M., Ramírez-Marcial, N., 2011.

Tropical montane cloud forests: current threats and opportunities for their conservation and sustainable management in Mexico. J. Environ. Manag. 92, 974–981. https://doi.org/10.1016/j.jenvman.2010.11.007.

Trujillo-Miranda, A.L., Toledo-Aceves, T., López-Barrera, F., Gerez-Fernandez, P., 2018. Active versus passive restoration: recovery of cloud forest structure, diversity and soil condition in abandoned pastures. Ecol. Eng. 117, 50–61. https://doi.org/10.1016/j.ecoleng.2018.03.011.

Urbina-Cardona, J.N., Londoño, M.C., 2003. Distribución de la comunidad de herpetofauna asociada a cuatro áreas con diferente grado de perturbación en la Isla Gorgona, Pacífico colombiano. Rev. Acad. Colomb. Cienc. 27, 105–113.

Urbina-Cardona, J.N., Bernal, E.A., Giraldo-Echeverry, N., Echeverry-Alcendra, A., 2015. El monitoreo de herpetofauna en los procesos de restauración ecológica: indicadores y métodos. In: Aguilar-Garavito, M., Ramírez, W. (Eds.), Monitoreo a procesos de restauración ecológica aplicado a ecosistemas terrestres. Instituto Humboldt, Colombia, pp. 134–147.

Venables, W.N., Ripley, B.D., 2002. Modern Applied Statistics. Springer, New York.

Vié, J.C., Hilton-Taylor, C., Stuart, S.N., 2009. Wildlife in a Changing World—An Analysis of the 2008 IUCN Red List of Threatened Species. IUCN, Gland, Switzerland.

Wake, D.B., Lynch, J.F., 1976. The Distribution, Ecology and Evolutionary History of Plethodontid Salamanders in Tropical America. Natural History Museum, USA.

Warnes, G.R., Bolker, B., Lumley, T., Johnson, R.C., 2018. gmodels: Various R Programming Tools for Model Fitting for R Version 2.18.1.

Wells, K.D., 2007. The Ecology and Behavior of Amphibians. The University of Chicago, Chicago.

Welsh, H.H., Hodgson, G.R., 2013. Woodland salamanders as metrics of forest ecosystem recovery: a case study from California's redwoods. Ecosphere 4, 1–25. https://doi.org/10.1890/ES12-00400.1.

Williams-Linera, G., 2002. Tree species richness complementarity, disturbance and fragmentation in a Mexican tropical montane cloud forest. Biodivers. Conserv. 1, 1825–1843.

Williams-Linera, G., 2015. El bosque mesófilo de montaña, veinte años de investigación ecológica ¿qué hemos hecho y hacia dónde vamos? Maderas y Bosques 21, 51–61.

WRI, 2019. The world lost a Belgium-sized area of primary rainforests last year. https://www.wri.org/blog/2019/04/world-lost-belgium-sized-area-primary-rainforests-last-year, Accessed date: 10 June 2019.

WWF, 2018. In: Grooten, M., Almond, R.E.A. (Eds.), Living Planet Report – 2018: Aiming Higher. WWF, Gland, Switzerland.

Glossary

Degraded ecosystem: an ecosystem that has partially lost its biodiversity and undergone simplification of its structure, composition and functionality (MEA 2005).

Ecological restoration: "process of assisting the recovery of an ecosystem that has been degraded, damaged, or destroyed" (SER, 2004).

Passive forest restoration: occurs when environmental stressors, e.g. grazing, are eliminated and secondary succession takes place naturally (Rey-Benayas et al. 2008).

Active forest restoration: occurs when the land is managed by planting vegetation, weeding, burning and/or thinning in order to achieve vegetation recovery (Rey-Benayas et al. 2008).

Recovery: "process by which an ecosystem regains its composition, structure and function relative to the levels identified for the reference ecosystem" (McDonald et al. 2016).

Reference ecosystem: "a community of organisms and abiotic components able to act as a model for restoration. A reference ecosystem represents a non-degraded version of the ecosystem complete with its flora, fauna, abiotic elements, functions and processes" (McDonald et al. 2016).

Hill numbers or effective numbers of species: a family of diversity indices that incorporate relative abundance and species richness. These indices differ among themselves only by an exponent N or q. To compare between samples, the first three Hill numbers are generally used: N_0 = species richness, N_1 = number of common species (exponential of Shannon's entropy index), and N_2 = number of dominant species (inverse of Simpson's concentration index; Hill, 1973; Chao et al., 2014).
