# Supplementary material for: Comparing the success of active and passive restoration in a tropical cloud forest landscape: A multi-taxa fauna approach
Source: PLoS One. 2020 Nov 10;15(11):e0242020. doi: 10.1371/journal.pone.0242020 (PMC7654786; doi:10.1371/journal.pone.0242020)
Supplement: S1 Appendix — P = cattle pasture, PR = forest under passive restoration, AR = forest under active restoration and CF = mature cloud forest. (DOCX) [file pone.0242020.s002.docx]

**S1 Appendix. Species of amphibians, ants and dung beetles recorded in four vegetation conditions, their abundance or occurrence frequency, and habitat specialization type. P = cattle pasture, PR = forest under passive restoration, AR = forest under active restoration and CF = mature cloud forest.**

| ID | Species | Vegetation conditions | | | | | Habitat  specialization |
| --- | --- | --- | --- | --- | --- | --- | --- |
|  |  |  | P | PR | AR | CF |  |
|  | **AMPHIBIANS** |  |  |  |  |  |  |
|  | Bufonidae |  |  |  |  |  |  |
| Iv | *Incilius valliceps* |  | 1 |  |  |  | Generalist^1,5,6,7^ |
|  | Craugastoridae |  |  |  |  |  |  |
| Cm | *Craugastor mexicanus* |  |  | 10 | 12 | 13 | Specialist^1^ |
| Cp | *Craugastor pygmaeus* |  | 8 | 16 | 19 | 14 | Generalist^1,3^ |
| Cr | *Craugastor rhodopis* |  | 9 | 88 | 113 | 57 | Specialist^1,2,5,6^ |
|  | Eleutherodactylidae |  |  |  |  |  |  |
| Ec | *Eleutherodactylus cystignathoides* |  | 4 | 3 | 2 | 1 | Generalist^1^ |
|  | Centrolenidae |  |  |  |  |  |  |
| Hv | *Hyalinobatrachium viridissimum* |  |  | 5 | 12 | 16 | Specialist^2,3^ |
|  | Hylidae |  |  |  |  |  |  |
| Ct | *Charadrahyla taeniopus* |  |  | 1 | 4 | 29 | Specialist^1,2,6^ |
| Rm | *Rheohyla miotympanum* |  | 41 | 26 | 38 | 29 | Generalist^1,3,5,6,7^ |
|  | Plethodontidae |  |  |  |  |  |  |
| Ac | *Aquiloeurycea cafetalera* |  |  | 5 | 7 | 20 | Specialist^1,2,4^ |
| Bp | *Bolitoglossa platydactyla* |  | 1 |  |  |  | Generalist^7^ |
| Pt | *Parvimolge townsendi* |  |  | 10 | 28 | 53 | Specialist^1,2^ |
| Tp | *Thorius pennatulus* |  |  |  | 3 | 7 | Specialist^4^ |
|  | Ranidae |  |  |  |  |  |  |
| Lb | *Lithobates berlandieri* |  | 8 |  |  |  | Generalist^1,6,7^ |
|  | **Species richness** |  | **7** | **9** | **10** | **10** |  |
|  | **Abundance** |  | **72** | **164** | **238** | **239** |  |
|  |  |  |  |  |  |  |  |
|  | **DUNG BEETLES** |  |  |  |  |  |  |
|  | Scarabaeidae |  |  |  |  |  |  |
| Ch | *Canthidium hespenheide* |  |  | 8 | 2 | 2 | Specialist^13,15^ |
| Cl | *Canthon leechi* |  | 7 |  |  |  | Generalist^10^ |
| Ci | *Copris incertus* |  | 30 |  |  |  | Generalist^8,9,11,12^ |
| Cc | *Coprophanaeus corythus* |  | 64 | 20 | 8 |  | Generalist^8,9,14^ |
| Cg | *Coprophanaeus gilli* |  |  | 7 | 36 | 5 | Specialist^9^ |
| Dm | *Deltochilum mexicanum* |  | 3 | 241 | 275 | 201 | Specialist^8,11,12,14^ |
| Dc | *Dichotomius colonicus* |  | 16 |  |  |  | Generalist^8,11,12^ |
| Ds | *Dichotomius satanas* |  | 72 | 64 | 71 | 54 | Generalist^8,9,12^ |
| Ob | *Onthophagus belorhinus* |  | 63 | 30 | 57 | 64 | Generalist^8,9^ |
| Oco | *Onthophagus corrosus* |  | 169 | 2 |  |  | Generalist^8,15^ |
| Oc | *Onthophagus cyanellus* |  | 1 | 12 | 5 | 230 | Specialist^8,11,12^ |
| Oi | *Onthophagus incensus* |  | 18 | 1 | 8 |  | Generalist^8,9,11,12,14,15^ |
| Or | *Onthophagus rhinolophus* |  |  | 2 |  | 1 | Specialist^8,12,14^ |
| Pe | *Phanaeus endymion* |  | 90 | 4 | 5 |  | Generalist^8,9,11,12,14^ |
| So | *Scatimus ovatus* |  | 6 |  |  |  | Generalist^8,12^ |
|  | **Species richness** |  | **13** | **11** | **9** | **7** |  |
|  | **Abundance** |  | **539** | **391** | **467** | **557** |  |
|  |  |  |  |  |  |  |  |
|  | **ANTS** |  |  |  |  |  |  |
|  | Formicidae |  |  |  |  |  |  |
| Ae | *Acromyrmex echinatior* |  | 3 | 3 |  |  | Generalist^23^ |
| Ap | *Adelomyrmex paratristani* |  |  |  |  | 1 | Generalist^18,19^ |
| As | *Adelomyrmex silvestrii* |  | 1 |  |  |  | Generalist^16,20^ |
| At | *Adelomyrmex tristani* |  |  | 15 | 13 | 16 | Specialist^16,18,19,20^ |
| Bo | *Brachymyrmex obscurior* |  |  |  | 10 | 6 | Generalist^23^ |
| Bsp | *Brachymyrmex* sp |  |  |  |  | 1 |  |
| Ca | *Camponotus abscisus* |  | 3 |  |  |  | Specialist^22^ |
| Cat | *Camponotus atriceps* |  | 15 | 4 | 6 | 1 | Generalist^16,17,19,20^ |
| Cc | *Camponotus claviscapus* |  |  |  |  | 1 | Specialist^16,17,20^ |
| Cs | *Camponotus striatus* |  | 1 | 2 | 1 | 1 | Specialist^17^ |
| Cr | *Cyphomyrmex rimosus* |  | 7 |  |  |  | Generalist^16,17,18^ |
| Db | *Dorymyrmex bicolor* |  | 1 |  |  |  | Generalist^16,17^ |
| Eb | *Eciton burchelli parvispinum* |  |  | 2 | 1 | 1 | Specialist^16,17,20,22^ |
| Fd | *Forelius damiani* |  | 1 |  |  |  | Generalist^17,20,22^ |
| Gs | *Gnamptogenys strigata* |  | 5 | 7 | 2 | 1 | Generalist^16,18,19,20,22^ |
| Hn | *Hypoponera nitidula* |  | 1 |  |  |  | Generalist^16^ |
| Lp | *Labidus praedator* |  |  |  | 2 |  | Generalist^16,18,19,20,22^ |
| Ld | *Linepithema dispertitum* |  | 2 |  |  | 2 | Specialist^23^ |
| Na | *Nylanderia austroccidua* |  | 2 | 4 | 6 | 8 | Specialist^20^ |
| Nb | *Nylanderia bourbonica* |  | 4 | 4 | 9 | 5 | Generalist^23^ |
| Nsp | *Nylanderia* sp |  |  |  |  | 2 |  |
| Ol | *Odontomachus laticeps* |  | 1 |  | 1 |  | Specialist^16^ |
| Pb | *Pheidole bilimeki* |  |  |  |  | 2 | Generalist^21^ |
| Pi | *Pheidole insipida* |  | 3 | 8 | 1 | 4 | Specialist^23^ |
| Pn | *Pheidole nubicola* |  | 6 | 1 |  | 4 | Specialist^16,17,18^ |
| Px | *Pheidole xyston* |  | 4 | 14 | 11 | 7 | Specialist^20^ |
| Pe | *Ponera exotica* |  |  |  |  | 2 | Generalist^20^ |
| Sg | *Solenopsis geminata* |  | 16 | 3 | 6 | 5 | Generalist^16,17,18,19,20,22^ |
| Ssp1 | *Solenopsis* sp 1 |  |  | 1 | 1 |  |  |
| Ssp2 | *Solenopsis* sp 2 |  |  | 1 |  |  |  |
| Sc | *Stenamma connectum* |  |  |  |  | 1 | Generalist^19^ |
| Se | *Stenamma excisum* |  |  | 1 | 5 |  | Specialist^19,20^ |
| Sf | *Stenamma felixi* |  |  |  |  | 1 | Specialist^20^ |
| Sv | *Stenamma vexator* |  |  | 1 | 1 | 5 | Specialist^18,19^ |
| Sb | *Strumigenys brevicornis* |  |  | 8 | 11 | 7 | Specialist^16,18,19^ |
| Sl | *Strumigenys ludia* |  |  |  | 1 | 2 | Specialist^16,17^ |
| Tr | *Tapinoma ramulorum* |  |  | 2 | 4 |  | Specialist^18^ |
| Tp | *Temnothorax* aff*. punctatissimus* |  | 5 |  | 1 | 3 | Specialist^23^ |
| Ts | *Trachymyrmex saussurei* |  |  |  | 3 | 2 | Specialist^23^ |
|  | **Species richness** |  | **19** | **18** | **21** | **26** |  |
|  | **Occurrence frequency** |  | **81** | **81** | **96** | **91** |  |

^1^ Murrieta-Galindo R, López-Barrera F, Gónzalez-Romero A, Parra-Olea G. Matriz and habitat quality in a montane cloud-forest landscape: amphibians in coffee plantations in central Veracruz, Mexico. Wild Res. 2013;40: 25-35. doi.org/10.1071/WR12076.

^2^ Meza-Parral Y, Pineda E. Amphibian diversity and threatened species in a severely transformed neotropical region in Mexico. PLOS ONE. 2015;10: e0121652. doi.org/10.1371/journal.pone.0121652.

^3^ Díaz-García JM, Pineda E, López-Barrera F, Moreno CE Amphibian species and functional diversity as indicators of restoration success in tropical montane forest. Biodivers Conserv. 2017;26: 2569-2589. doi.org/10.1007/s10531-017-1372-2.

^4^ Sandoval-Comte A, Pineda E, Aguilar-López JL. In search of critically endangered species: the current situation of two tiny salamanders species in the Neotropical mountains of Mexico. PLOS ONE. 2012;7: e34023. doi.org/10.1371/journal.pone.0034023.

^5^ Lara-Tufiño JD, Badillo-Saldaña LM, Hernández-Austria R, Ramírez-Bautista A. Effects of traditional agroecosystems and grazing areas on amphibian diversity in a region of central Mexico. PeerJ. 2019;7: e6390. doi.org/10.7717/peerj.6390

^6^ Badillo-Saldaña LM, Ramírez-Bautista A, Wilson LD. Effects of establishment of grazing areas on diversity amphibian communities in tropical evergreen forests and mountain cloud forests of the Sierra Madre Oriental. Rev Mex Biodivers. 2016;87: 133-139.

^7^ Cruz-Elizalde R, Berriozabal-Islas C, Hernández-Salinas U, Martínez-Morales MA, Ramírez-Bautista A. Amphibian species richness and diversity in a modified tropical environment of central Mexico. J Trop Ecol. 2016;57: 407-417.

^8^ Huerta CC, Arellano GL, Cruz RM, Escobar SF, Martínez MI. Los escarabajos del estiércol en los potreros ganaderos de Xico. Instituto de Ecología, A. C. Xalapa, Veracruz, México. 2016.

^9^ Deloya C, Parra-Tabla V, Delfín-González H. Fauna de Coleópteros Scarabaeidae, Laparosticti y Trogidae (Coleoptera: Scarabaeoidea) asociados al bosque mesófilo de montaña, cafetales bajo sombra y comunidades derivadas del centro de Veracruz, México. Neotrop Entomol. 2007;36: 5-21. doi.org/10.1590/S1519-566X2007000100002

^10^ Rivera-Cervantes LE, Halffter G. Monografía de las especies mexicanas de Canthon del subgénero Glaphyrocanthon (Coleoptera: Scarabaeidae: Scarabaeinae). Acta Zool Mex. 1999;77: 23-150.

^11^ Arellano L, Favila ME, Huerta C. Diversity of dung and carrion beetles in a disturbed Mexican tropical montane cloud forest and on shade coffee plantations. Biodivers Conserv 2005;14: 601-615. doi.org/10.1007/s10531-004-3918-3

^12^ Pineda E, Moreno C, Escobar F, Halffter G. Frog, Bat, and Dung Beetle Diversity in the Cloud Forest and Coffee Agroecosystems of Veracruz, Mexico. Conserv Biol. 2005;19: 400-410. doi.org/ 10.1111/j.1523-1739.2005.00531.x

^13^ Chamorro W, Marin-Armijos D, Senjo A, Vaz de Mello FZ. Scarabaeinae dung beetles from Ecuador: a catalog, nomenclatural acts, and distribution records. ZooKeys. 2019;826: 1-343. doi.org/10.3897/zookeys.826.26488

^14^ Sánchez-Hernández G, Gómez B, Delgado L, Rodríguez-López ME, Chamé-Vázquez ER. Diversidad de escarabajos copronecrófagos (Coleoptera: Scarabaeidae: Scarabaeinae) en la Reserva de la Biosfera Selva El Ocote, Chiapas, México. Caldasia. 2018;40: 144 160.

doi.org/10.15446/caldasia.v40n1.68602

^15^ Santiago-Molina JP, Chamorro-Florescano IA, Amézquita-Melo SJ, Pech-Canché JM. Escarabajos copro-necrófagos (Coleoptera: Scarabaeinae) en fragmentos de selva baja caducifolia, pastizales y cultivos de vainilla en San Lorenzo Tajín, Papantla, Veracruz, México. Revista Biológico Agropecuaria Tuxpan. 2014;2: 842-852.

^16^ Valenzuela-González J, Quiroz-Robledo L, Martínez-Tlapa DL. Hormigas (Insecta: Hymenoptera: Formicidae). In Manson RH, Hernández-Ortiz V, Gallina S, Mehltreter K, editors. Agroecosistemas cafetaleros de Veracruz: biodiversidad, manejo y conservación.. Instituto de Ecología A. C., México; 2008. pp. 107-122.

^17^ García-Martínez MÁ, Martínez-Tlapa DL, Pérez-Toledo GR, Quiroz-Robledo LN, Castaño-Meneses G, Laborde J, Valenzuela-González JE. Taxonomic species and functional group diversity of ants in a tropical anthropogenic landscape. Trop Conserv Sci. 2015;8: 1017-1032.

doi.org/10.1177/194008291500800412

^18^ Pérez-Toledo GR, Valenzuela-Gónzalez JE, Flores-Galván C, Gallardo-Hernández C, Vásquez-Torres V, García-Martínez MA. Hormigas (Hymenoptera: Formicidae) asociadas a tres tipos de vegetación de un paisaje agropecuario en Veracruz. Entomología mexicana. 2016;3: 582-588

^19^ García-Martínez MÁ, Valenzuela-González JE, Escobar-Sarria F, López-Barrera F, Castaño-Meneses G. The surrounding landscape influences the diversity of leaf-litter ants in riparian cloud forest remnants. PLOS ONE 2017;12: e0172464. doi.org/10.1371/journal.pone.0172464

^20^ García-Martínez MA, Martínez-Tlapa DL, Pérez-Toledo GR, Quiroz-Robledo LN, Valenzuela-González JE. Myrmecofauna (Hymenoptera: Formicidae) response to hábitat characteristics of tropical montane cloud forest in central Veracruz, Mexico. Fla Entomol. 2016;99: 248-256. doi.org/10.1653/024.099.0214

^21^ García-Martínez MA, Vanoye-Eligio V, Leyva-Ovalle OR, Zetina-Córdoba P, Aguilar-Méndez MJ, Rosas-Mejía M. Diversity of ants (Hymenoptera: Formicidae) in a sub-montane and sub-tropical cityscape of northeastern Mexico. Sociobiology. 2019;66: 440-447. doi.org/ 10.13102/sociobiology.v66i3.4264

^22^ Landero-Torres I, Madrid-Ñeco I, Valenzuela-González JE, Galindo-Tovar ME, Leyva-Ovalle OR, Murguía-González J, Lee-Espinosa HE, García-Martínez MA. Myrmecofauna from three ornamental agroecosystems with different management and a forest remnant in Ixtaczoquitlán, Veracruz, México. Southwestern Entomol. 2014;39: 783-795. doi.org/10.3958/059.039.0409

^23^ AntWeb. Version 8.16.2. California Academy of Science, online at https://www.antweb.org. Accessed 19 March 2020.
