# Supplementary material for: Comparing the success of active and passive restoration in a tropical cloud forest landscape: A multi-taxa fauna approach
Source: PLoS One. 2020 Nov 10;15(11):e0242020. doi: 10.1371/journal.pone.0242020 (PMC7654786; doi:10.1371/journal.pone.0242020)
Supplement: S2 Appendix — Mean and standard deviation (in parentheses) values are presented for all variables. Environmental variables considered in models to predict responses of each group are indicated with an X. NC = not considered in models due to high Pearson correlation coefficients (≥ 0.6, p < 0.05). (DOCX) [file pone.0242020.s003.docx]

**S2 Appendix. Environmental variables in the four vegetation conditions studied: P = cattle pasture, PR = 23-year-old forest under passive restoration, AR = 23-year-old forest under active restoration and CF = mature cloud forest. Mean and standard deviation (in parentheses) values are presented for all variables. Habitat variables considered in models to predict responses of each group are indicated with an X. NC = not considered in models due to high Pearson correlation coefficients (≥ 0.6, p < 0.05).**

|  | **Vegetation types** | | | | **Faunal group** | | | |
| --- | --- | --- | --- | --- | --- | --- | --- | --- |
|  | **P** | **PR** | **AR** | **CF** | **Amphibians** | **Ants** | **Dung beetles** |  |
| Distance to stream (m) | 182.5 (131.0) | 87.3 (58.7) | 78.3 (55.2) | 60.1 (25.8) | X |  |  |  |
| Distance to CF (m) | 1800.6 (205) | 1152 (298.0) | 995.1 (322.0) | 0 (0) | X | X | X |  |
| Tree density (trees/ha) | 16.6 (35.5) | 383 (141.2) | 461.1(89.4) | 588.9 (193.0) | X | X | X |  |
| Canopy cover (%) | 13.1 (15.3) | 72.3 (5.0) | 76.1 (6.0) | 77.4 (3.7) | X | X | X |  |
| Leaf litter cover (%) | 7.7 (8.8) | 44.2 (20.8) | 50.3 (24.2) | 67.8 (13.6) | X | X | X |  |
| Bare soil cover (%) | 4.7 (10.0) | 6 (5.7) | 2.7 (2.6) | 12 (10.0) | NC | NC | NC |  |
| Exotic grasses cover (%) | 84.7 (23.1) | 15.8 (8.7) | 11.6 (10.0) | 3.5 (7.7) | NC | NC | NC |  |
| Cover of non-grass herbaceous plants (%) | 2.8 (6.8) | 34 (22.0) | 35.4 (26.6) | 16.6 (6.4) | X | X | X |  |
| Other shrubs cover (%) | 1 (2.5) | 20.3 (11.2) | 15.1 (6.8) | 30.8 (13.1) | NC | NC | NC |  |
| Cover of Piperaceae shrubs (%) | 0 (0) | 9.5 (10.8) | 12.4 (15.3) | 1.5 (4.4) | NC | NC | NC |  |
| Cover of *Pteridium* *arachnoideum* (%) | 0 (0) | 5 (9.9) | 1 (1.0) | 1 (1.0) | X | X | X |  |
| Epiphyte cover (%) | 7.3 (11.4) | 9.5 (12.2) | 11.1 (8.7) | 49 (25.5) | X | X |  |  |
| Number of fallen trunks | 0.7 (1.25) | 2.1 (1.5) | 3.2 (1.8) | 5.6 (1.6) | X | X |  |  |
| Soil compaction (Kg/cm^2^) | 2.3 (0.5) | 1.4 (0.5) | 1.1 (0.7) | 0.6 (0.3) |  | X | X |  |
