# Supplementary material for: Comparing the success of active and passive restoration in a tropical cloud forest landscape: A multi-taxa fauna approach
Source: PLoS One. 2020 Nov 10;15(11):e0242020. doi: 10.1371/journal.pone.0242020 (PMC7654786; doi:10.1371/journal.pone.0242020)
Supplement: S3 Appendix — Results of generalized linear models used to compare the abundance of amphibians and dung beetles, and results of goodness-of-fit Chi-square tests used to compare the occurrence-frequency of ants. P = cattle pasture, PR = 23-year-old forest under passive restoration, AR = 23-year-old forest under active restoration and CF = mature cloud forest. Table 2. Results of post hoc tests comparing the abundance of amphibians and dung beetles, and the occurrence-frequency of ants. P = cattle pasture, PR = 23-year-old forest under passive restoration, AR = 23-year-old forest under active restoration and CF = mature cloud forest. In all comparisons the degrees of freedom = 1. (DOCX) [file pone.0242020.s004.docx]

**S3 Appendix. Table 1. Results of generalized linear models used to compare the abundance of amphibians and dung beetles, and results of goodness-of-fit Chi-square tests used to compare the occurrence frequency of ants. P = cattle pasture, PR = 23-year-old forest under passive restoration, AR = 23-year-old forest under active restoration and CF = mature cloud forest.**

|  | **Statistics** |
| --- | --- |
| **Amphibians** |  |
| Total | Deviance = 145.7, d. f. = 32, *P* < 0.001 |
| Forest specialist | Deviance = 238.9, d. f. = 32, *P* < 0.001 |
| Generalist | Deviance = 5.3, d. f. = 32, *P* = 0.1 |
|  |  |
| **Dung beetles** |  |
| Total | Deviance = 36.1, d. f. = 32, *P* < 0.001 |
| Forest specialist | Deviance = 592.8, d. f. = 32, *P* < 0.001 |
| Generalist | Deviance = 454.9, d. f. = 32, *P* < 0.001 |
|  |  |
| **Ants** |  |
| Total | X^2^ = 1.9, d. f. = 7, *P* = 0.9 |
| Forest specialist | X^2^ = 15.6, d. f. = 7, *P* < 0.01 |
| Generalist | X^2^ = 18.9, d. f = 7, *P* < 0.01 |

S3. Table 2. Results of *post hoc* tests comparing the abundance of amphibians and dung beetles, and the occurrence frequency of ants. P = cattle pasture, PR = 23-year-old forest under passive restoration, AR = 23-year-old forest under active restoration and CF = mature cloud forest. In all comparisons the degrees of freedom = 1

|  | **Amphibians** | | |
| --- | --- | --- | --- |
|  | Total | Forest specialists | Generalists |
| P vs. PR | X^2^ = 45.9,  *P* < 0.001 | X^2^ = 55.8,  *P* < 0.001 |  |
| P vs. AR | X^2^ = 13.5,  *P* < 0.001 | X^2^ = 76.6,  *P* < 0.001 |  |
| P vs. CF | X^2^ = 79.1,  *P* < 0.001 | X^2^ = 81.4,  *P* < 0.001 |  |
| PR vs. AR | X^2^ = 10.9,  *P* < 0.001 | X^2^ = 11.9,  *P* < 0.001 |  |
| PR vs. CF | X^2^ = 17.5,  *P* < 0.001 | X^2^ = 18.1,  *P* < 0.001 |  |
| AR vs. CF | X^2^ = 0.01,  *P* = 0.91 | X^2^ = 0.7,  *P* = 0.41 |  |
|  | **Dung beetles** | | |
|  | Total | Forest specialists | Generalists |
| P vs. PR | X^2^ = 23.3,  *P* < 0.001 | X^2^ = 69.9  *P* < 0.001 | X^2^ = 218.1,  *P* < 0.001 |
| P vs. AR | X^2^ = 5.1,  *P* < 0.05 | X^2^ = 75.6,  *P* < 0.001 | X^2^ = 190.4,  *P* < 0.001 |
| P vs. CF | X^2^ = 0.3,  *P* = 0.58 | X^2^ = 87.5,  *P* < 0.001 | X^2^ = 220.9,  *P* < 0.001 |
| PR vs. AR | X^2^ = 6.7,  *P* < 0.01 | X^2^ = 3.9,  *P* < 0.05 | X^2^ = 2.9,  *P* = 0.08 |
| PR vs. CF | X^2^ = 28.7,  *P* < 0.001 | X^2^ = 39.5,  *P* < 0.001 | X^2^ = 0.1,  *P* = 0.84 |
| AR vs. CF | X^2^ = 7.9,  *P* < 0.01 | X^2^ = 19.17,  *P* < 0.001 | X^2^ = 3.6,  *P* = 0.06 |
|  |  |  |  |
|  | **Ants** | | |
|  | Total | Forest specialists | Generalists |
| P vs. PR |  | raw.p = 0.018,  *P* < 0.05 | raw.p = 0.009,  *P* < 0.05 |
| P vs. AR |  | raw.p = 0.012,  *P* < 0.05 | raw.p = 0.25,  *P* < 0.05 |
| P vs. CF |  | raw.p = 0.005,  *P* < 0.05 | raw.p = 0.02,  *P* < 0.05 |
| PR vs. AR |  | raw.p = 0.99,  *P* = 0.9 | raw.p = 0.21,  *P* = 0.33 |
| PR vs. CF |  | raw.p = 0.78,  *P* = 0.9 | raw.p = 0.84,  *P* = 0.84 |
| AR vs. CF |  | raw.p = 0.89,  *P* = 0.9 | raw.p = 0.36,  *P* = 0.44 |
